# Supplementary material for: AI-powered spatial cell phenomics enhances risk stratification in non-small cell lung cancer
Source: Nat Commun. 2025 Nov 3;16:9701. doi: 10.1038/s41467-025-65783-z (PMC12583542; doi:10.1038/s41467-025-65783-z)
Supplement: Supplementary file 1 — Supplementary Information [file 41467_2025_65783_MOESM1_ESM.pdf]

Supplementary Information

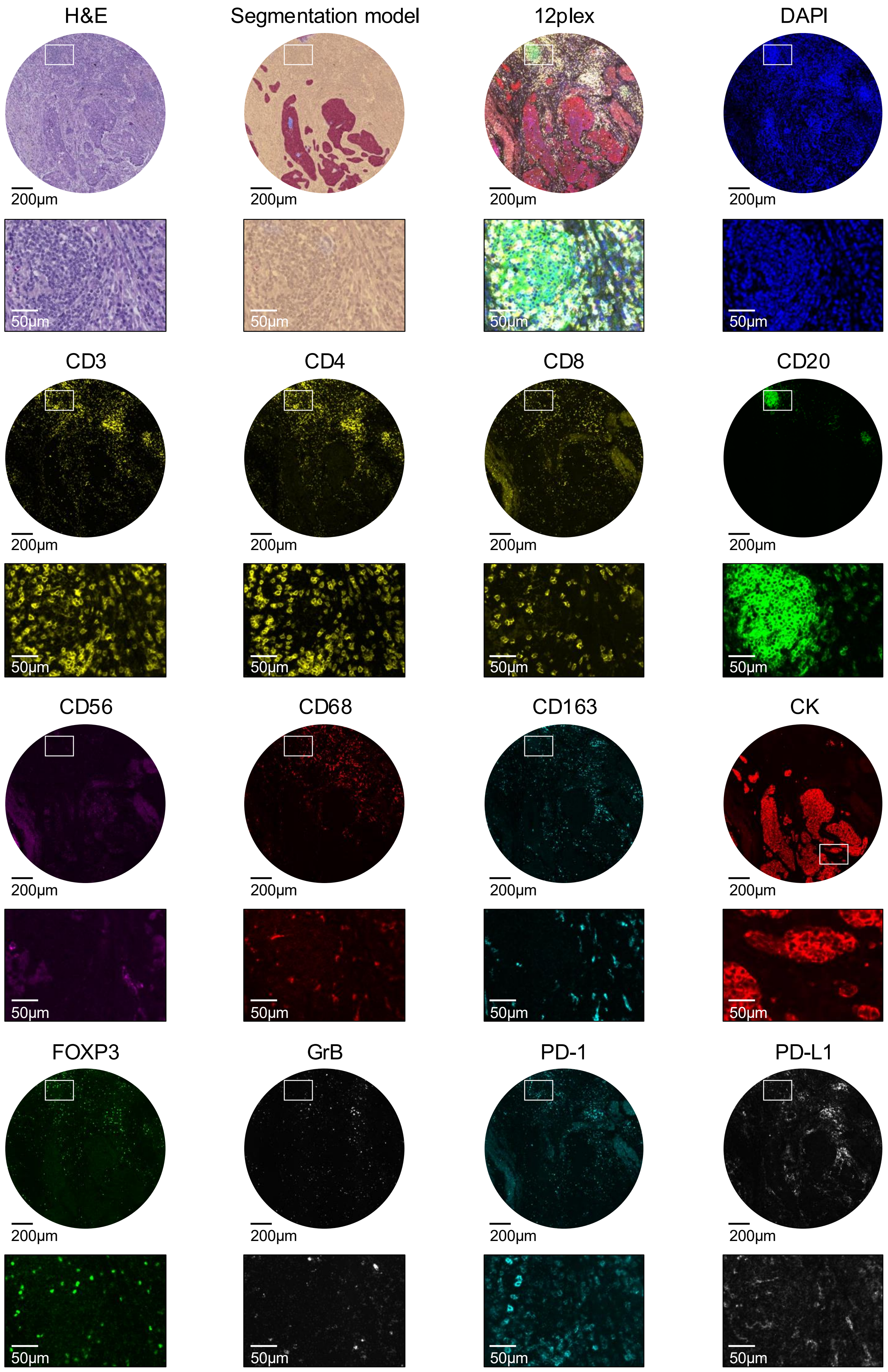

Supplementary Figure 1: Overview of imaging data modalities and markers.

Each of the four rows shows different image modalities and marker views of the same representative tumor sample (n = 1), with whole tissue spots in the upper panel and its respective magnified views directly below. From left to right in the top row: H&E staining, tissue segmentation model heatmap, the combined 12-plex mIF panel, and the DAPI channel (blue). The second row shows CD3, CD4, and CD8 (all in yellow) and CD20 (green). The third-row displays CD56 (magenta), CD68 (red), CD163 (cyan-blue), and CK (red). The fourth row shows FOXP3 (green), GrB (white), PD-1 (cyan-blue), and PD-L1 (white).

Images are read from left to right in each row. Scale bars: 200μm (tissue spots) and 50μm (magnified regions).

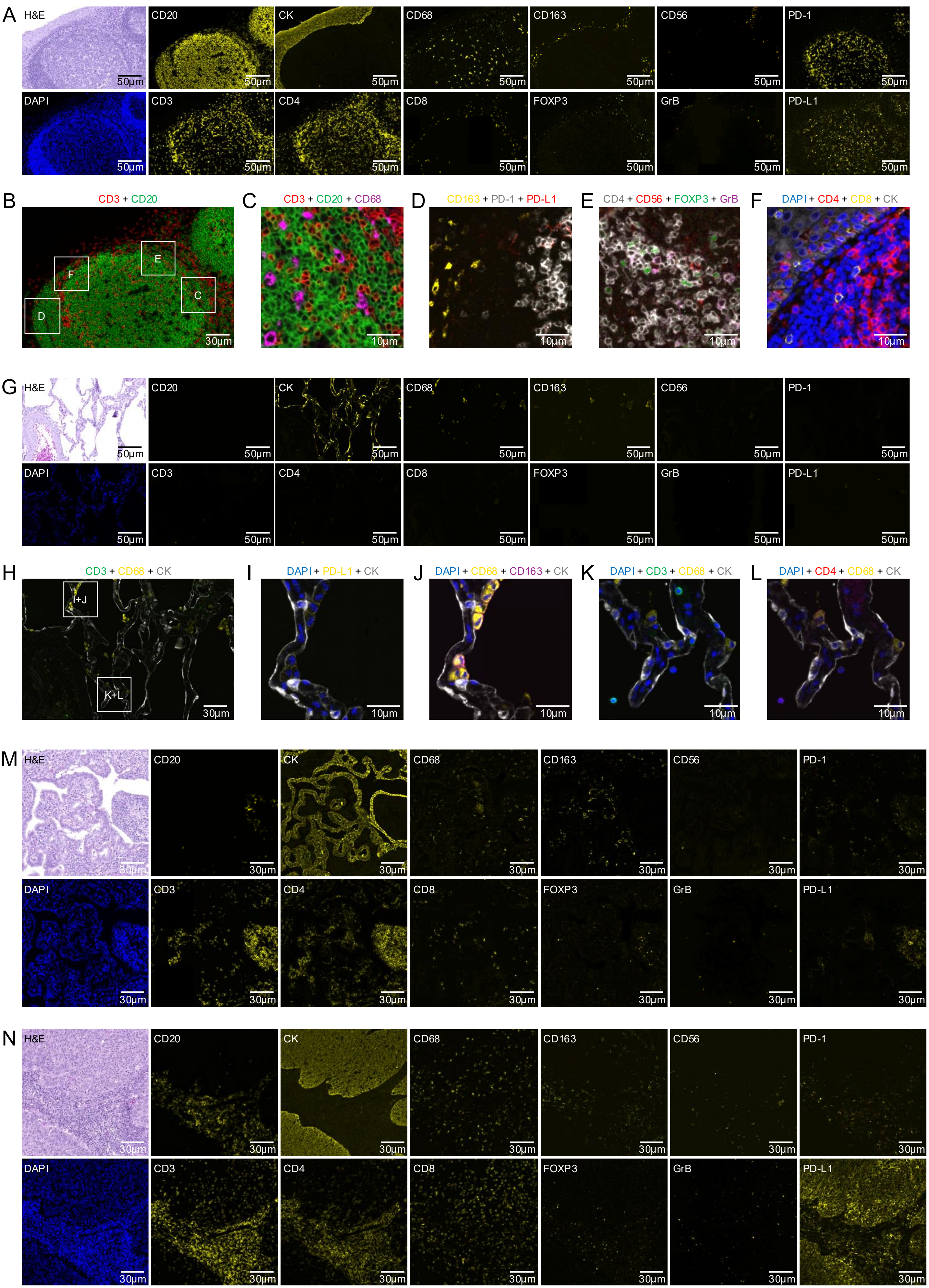
A
H&E
CD20
CK
CD68
CD163
CD56
PD-1
DAPI
CD3
CD4
CD8
FOXP3
GrB
PD-L1
50µm
B
CD3 + CD20
F
E
C
D
30µm
C
CD3 + CD20 + CD68
10µm
D
CD163 + PD-1 + PD-L1
E
CD4 + CD56 + FOXP3 + GrB
F
DAPI + CD4 + CD8 + CK
G
H&E
CD20
CK
CD68
CD163
CD56
PD-1
DAPI
CD3
CD4
CD8
FOXP3
GrB
PD-L1
H
CD3 + CD68 + CK
I+J
K+L
I
DAPI + PD-L1 + CK
J
DAPI + CD68 + CD163 + CK
K
DAPI + CD3 + CD68 + CK
L
DAPI + CD4 + CD68 + CK
M
H&E
CD20
CK
CD68
CD163
CD56
PD-1
DAPI
CD3
CD4
CD8
FOXP3
GrB
PD-L1
N
H&E
CD20
CK
CD68
CD163
CD56
PD-1
DAPI
CD3
CD4
CD8
FOXP3
GrB
PD-L1

Supplementary Figure 2: Marker validation.

(A) Validation of markers on lymphatic tissue (n = 1 tissue sample): The tonsil tissue shows the expected staining patterns for all applied markers.

(B-F) Staining overlays of tonsil tissue (n = 1 tissue sample) demonstrate the expected patterns of histologic architecture, subcellular structures (nucleus, cytoplasm, membrane), and protein co-expression.

(G) Validation of the assay on lung tissue (n = 1 tissue sample) : Lung tissue shows the expected staining patterns for all applied markers. Staining artifacts, which are frequently observed in erythrocytes (bottom left) and within the alveolar spaces, are not visible.

(H-L) Staining overlays of lung tissue (n = 1 tissue sample) illustrate the expected patterns of histologic architecture, subcellular structures (nucleus, cytoplasm, membrane), and protein co-expression.

(M+N) Representative staining of one LUAD (n = 1 tumor sample; M) and one LUSC patient (n = 1 tumor sample; N).

Scale bars: (A+G) 50µm, (B+H+M+N) 30µm, (C-F+I-L) 10µm

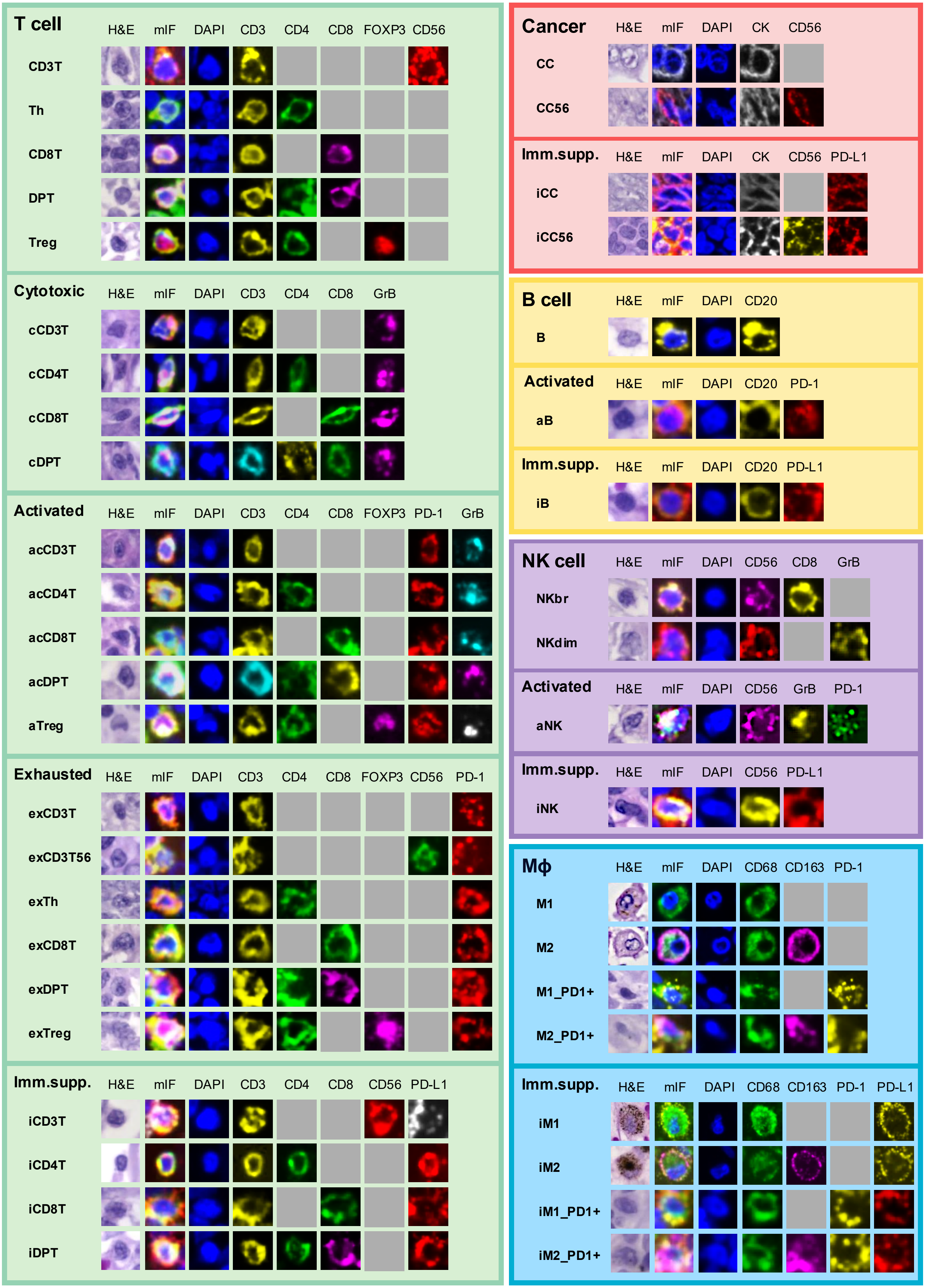
T cell
H&E mIF DAPI CD3 CD4 CD8 FOXP3 CD56
CD3T
Th
CD8T
DPT
Treg
Cytotoxic
H&E mIF DAPI CD3 CD4 CD8 GrB
cCD3T
cCD4T
cCD8T
cDPT
Activated
H&E mIF DAPI CD3 CD4 CD8 FOXP3 PD-1 GrB
acCD3T
acCD4T
acCD8T
acDPT
aTreg
Exhausted
H&E mIF DAPI CD3 CD4 CD8 FOXP3 CD56 PD-1
exCD3T
exCD3T56
exTh
exCD8T
exDPT
exTreg
Imm.supp.
H&E mIF DAPI CD3 CD4 CD8 CD56 PD-L1
iCD3T
iCD4T
iCD8T
iDPT
Cancer
H&E mIF DAPI CK CD56
CC
CC56
Imm.supp.
H&E mIF DAPI CK CD56 PD-L1
iCC
iCC56
B cell
H&E mIF DAPI CD20
B
Activated
H&E mIF DAPI CD20 PD-1
aB
Imm.supp.
H&E mIF DAPI CD20 PD-L1
iB
NK cell
H&E mIF DAPI CD56 CD8 GrB
NKbr
NKdim
Activated
H&E mIF DAPI CD56 GrB PD-1
aNK
Imm.supp.
H&E mIF DAPI CD56 PD-L1
iNK
Mϕ
H&E mIF DAPI CD68 CD163 PD-1
M1
M2
M1_PD1+
M2_PD1+
Imm.supp.
H&E mIF DAPI CD68 CD163 PD-1 PD-L1
iM1
iM2
iM1_PD1+
iM2_PD1+

Supplementary Figure 3: Marker expression across all 43 cell phenotypes.
Cell phenotypes are shown for one representative tumor sample (n = 1), classified according to their primary cell type and subsequently subdivided based on their cellular status (cytotoxic, activated, exhausted, immunosuppressive). T cells are indicated by green, carcinoma cells by red, B cells by yellow, NK cells by purple and macrophages by cyan. Figure shows cell phenotype name abbreviations. For full names see Supplementary Table 2.
Scale: each image represents a 15 µm field of view.

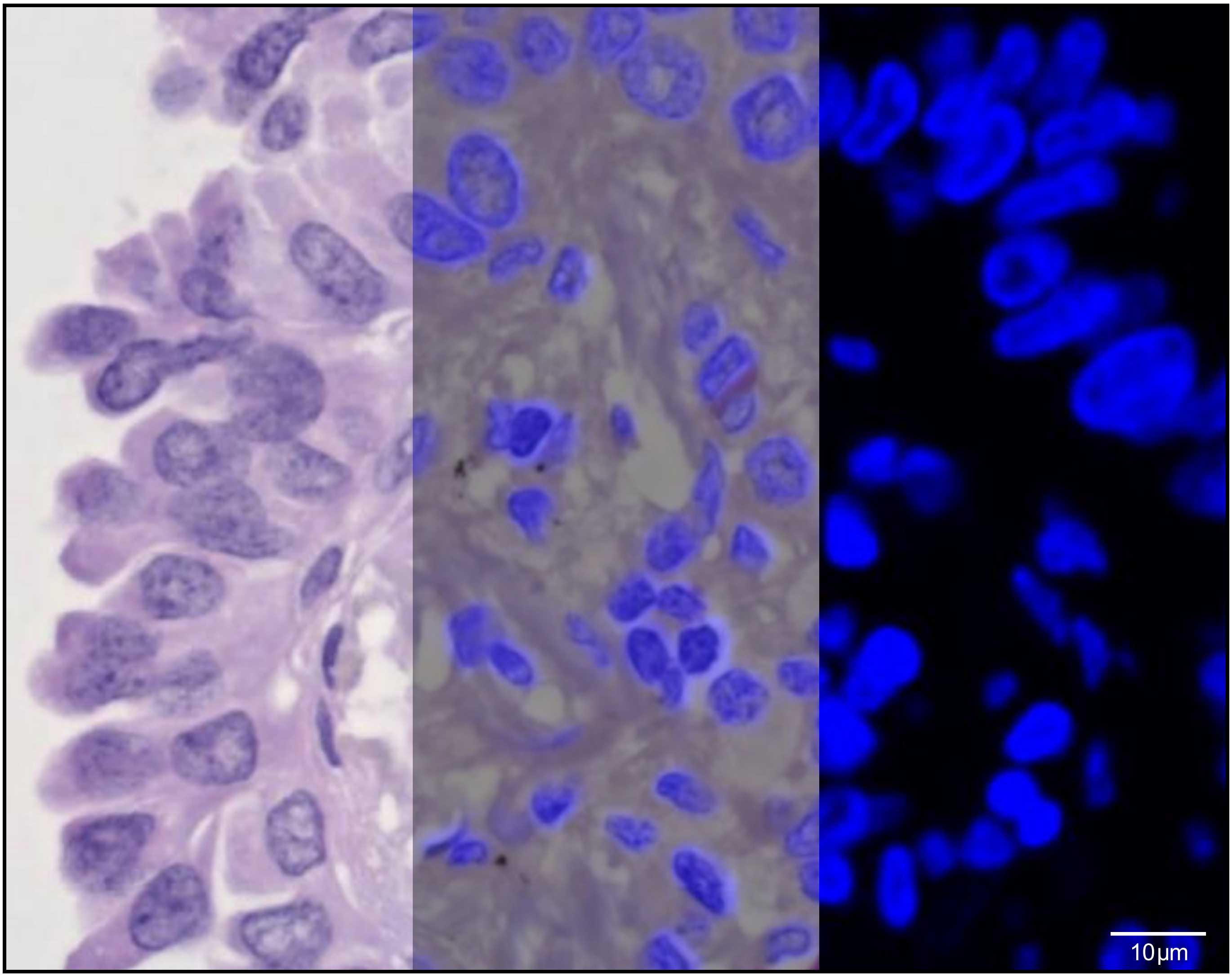

Supplementary Figure 4: Co-registration of H&E and mIF data.
Center image showing precise alignment of H&E-stain (left) and mIF (DAPI) stain (right; n = 1 tumor sample).
Scale bar: 10μm

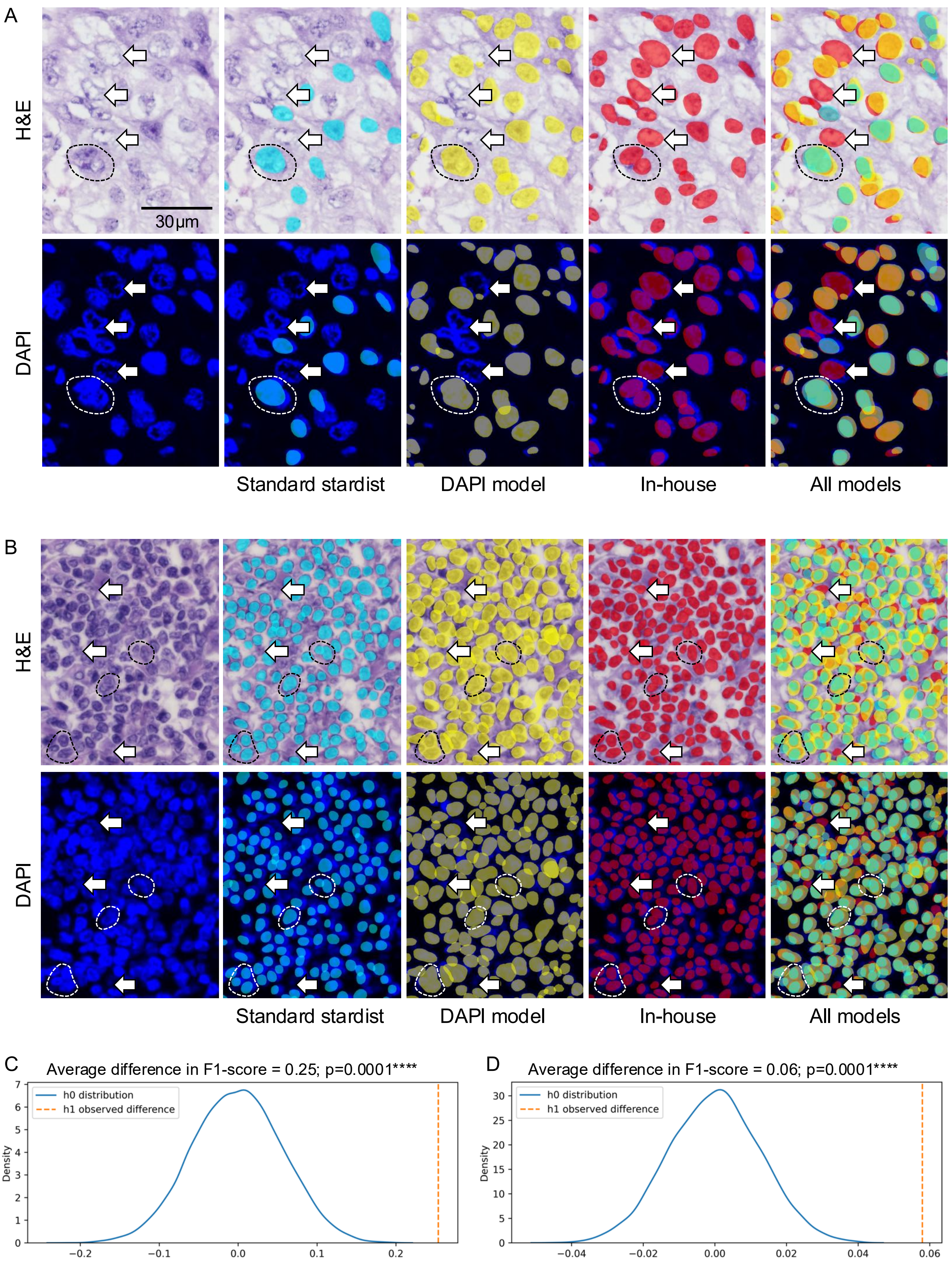
A
H&E
30μm
DAPI
Standard stardist
DAPI model
In-house
All models
B
H&E
DAPI
Standard stardist
DAPI model
In-house
All models
C
Average difference in F1-score = 0.25; p=0.0001****
h0 distribution
h1 observed difference
Density
D
Average difference in F1-score = 0.06; p=0.0001****
h0 distribution
h1 observed difference
Density

Supplementary Figure 5: Comparative analysis of cell detection models.
Standard StarDist model (cyan; F1-score=0.682), standard DAPI model (yellow; F1-score=0.855) compared to our optimized StarDist model (red; F1-score=0.913).
(A) The standard models are less sensitive (recall) and miss to detect cells that our optimized version does cover (exemplary cells are indicated by white arrows; n = 1 tumor sample). In comparison to our optimized model, individual cells are less well discriminated by the standard models (two exemplary cells framed by white dashed lines are detected as a single cell by the standard models).
(B) Similar observations can be made within tissue areas of high cellularity (n = 1 tumor sample). In particular, the DAPI model often does not discriminate well between individual cells (exemplary cells framed by white dashed lines).
(C) The performance of the In-house model is compared to the Standard StarDist (H&E) model and tested for statistical significance using a permutation test over the per slide F1 scores.
(D) Same as (C) but for comparison of In-house and the DAPI model.
Scale bars: (A+B) 30µm

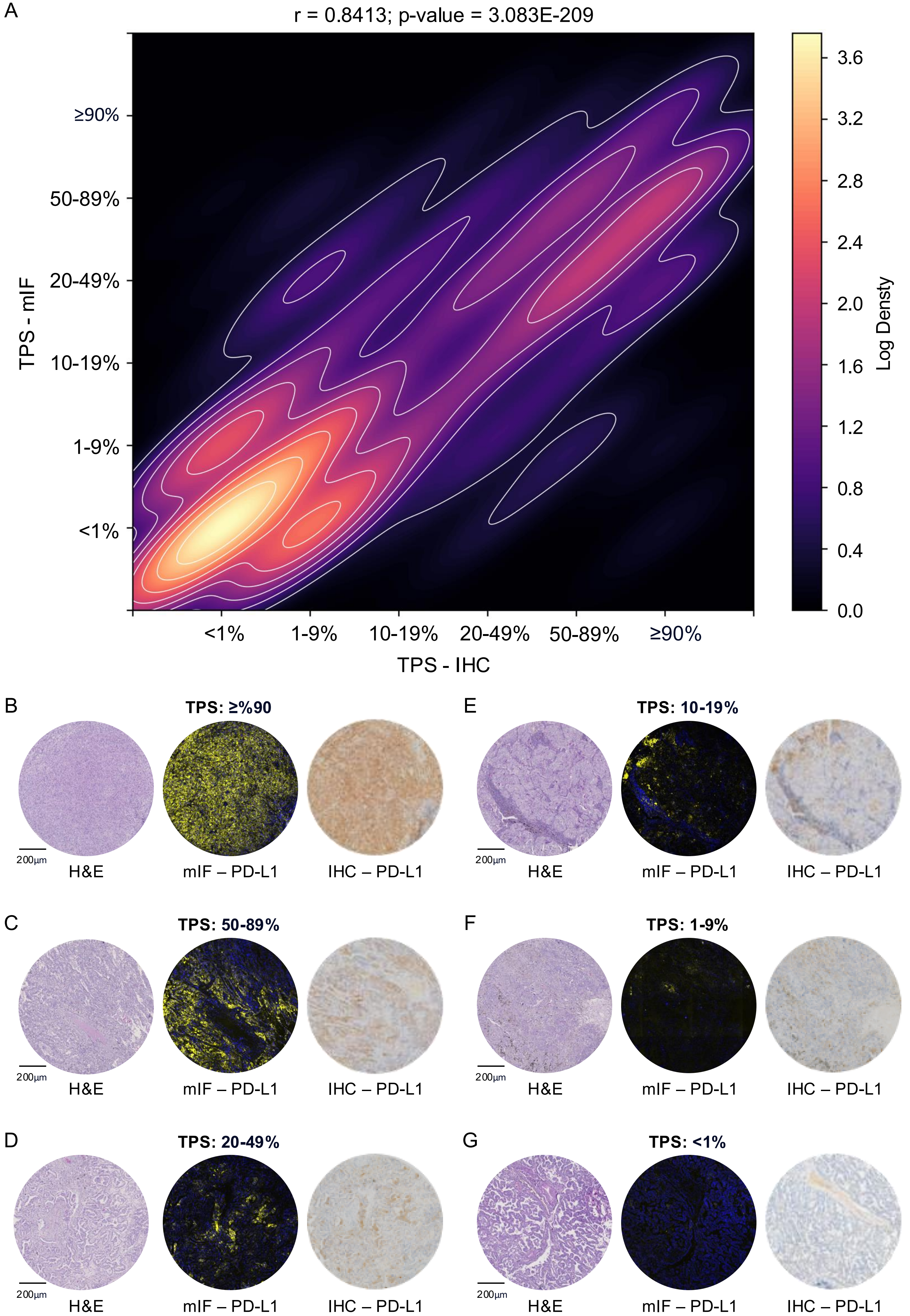

A
r = 0.8413; p-value = 3.083E-209
TPS - mIF
≥90%
50-89%
20-49%
10-19%
1-9%
<1%
TPS - IHC
Log Density
3.6
3.2
2.8
2.4
2.0
1.6
1.2
0.8
0.4
0.0
B
TPS: ≥%90
C
TPS: 50-89%
D
TPS: 20-49%
E
TPS: 10-19%
F
TPS: 1-9%
G
TPS: <1%
200µm
H&E
mIF – PD-L1
IHC – PD-L1

Supplementary Figure 6: Validation of the tumor proportion score (TPS) by IHC.
(A) Correlation of the TPS evaluation through automated analysis of mIF staining (y-axis), with the evaluation through the pathologist of IHC staining (x-axis). Statistical significance was assessed using Pearson's correlation (scipy.stats.pearsonr; n = 769 patients).
(B-G) Examples of tumors with different TPS ranges, stained by H&E (left), mIF (center) and IHC (right).
Colors in mIF: Blue = DAPI, yellow = PD-L1.
Scale bars: (B-G) 200µm

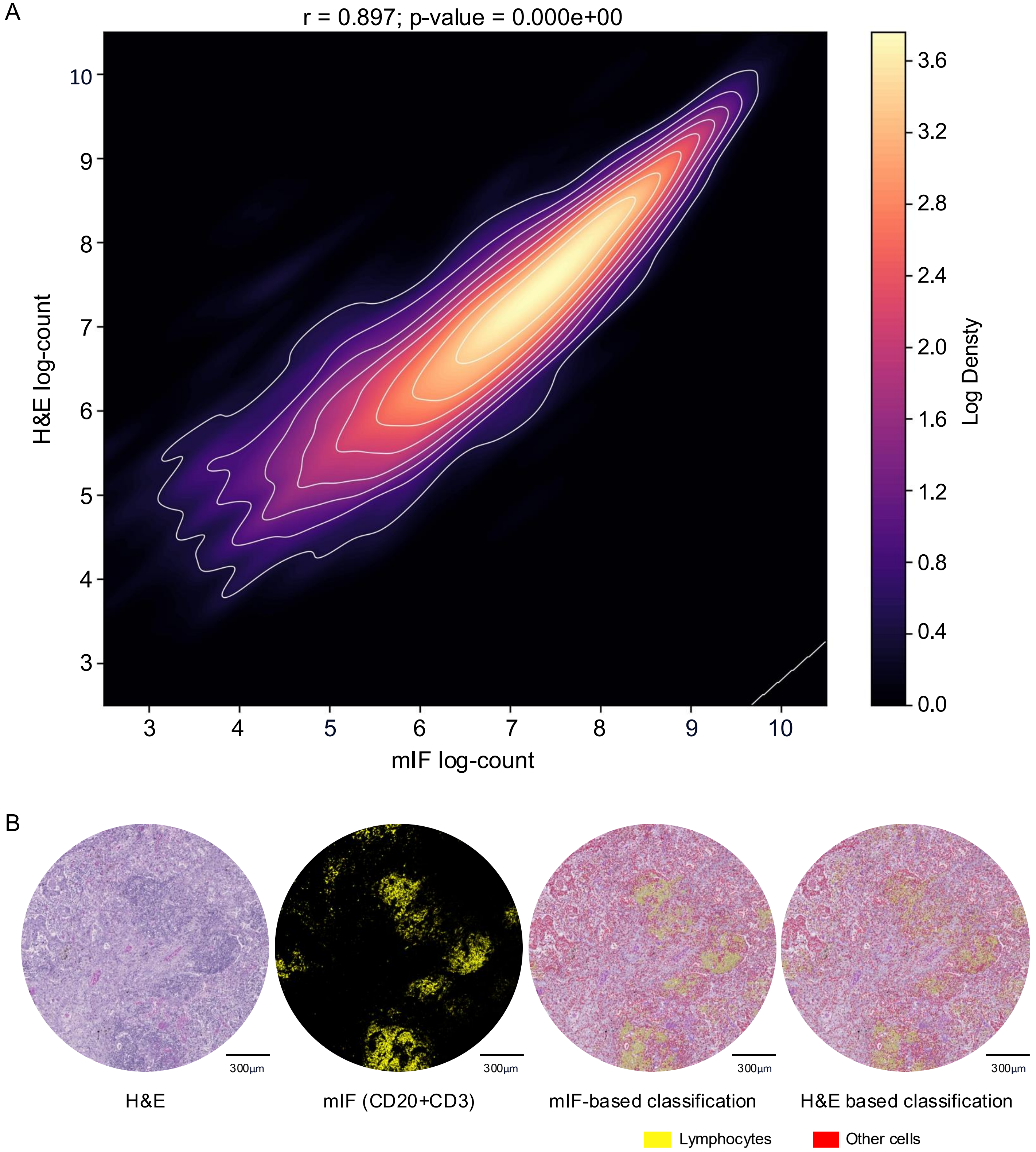

Supplementary Figure 7: Validation of the lymphocyte counts by H&E-based cell classification.
(A) Correlation of the lymphocytes counts detected by H&E-based cell classification (y-axis), with detection by mIF-based cell classification (x-axis). Statistical significance was assessed using Pearson's correlation (scipy.stats.pearsonr; n = 3807 tumor samples from 1125 patients).
(B) Exemplary tissue core stained by H&E (left), stained by mIF (center left), with AI-derived mIF-based cell classification (center right) and AI-derived H&E-based cell classification (right). Colors in mIF: Yellow = CD3+CD20.
Scale bars: (B) 300µm

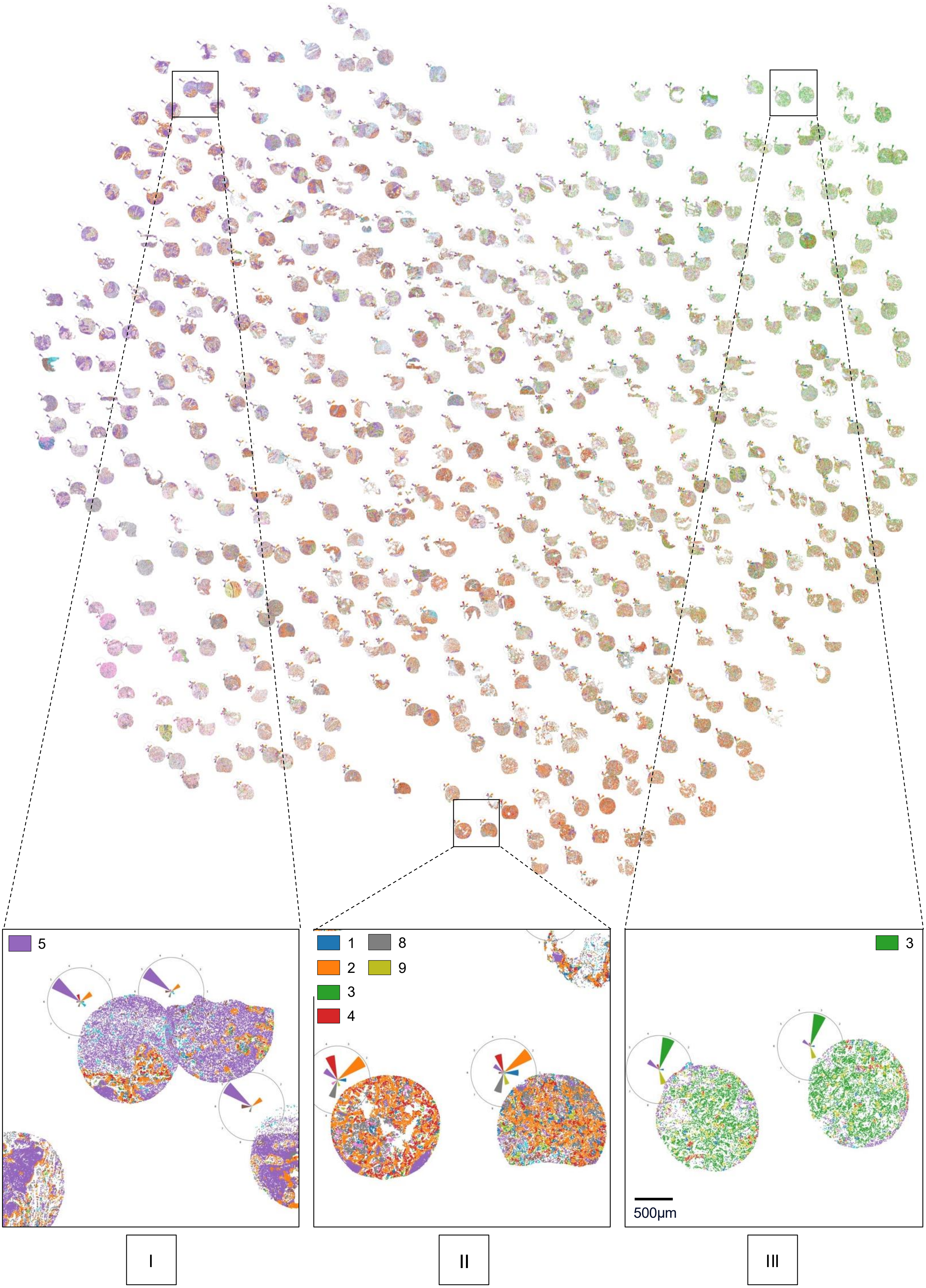

5
1
8
2
9
3
4
3
500μm
I
II
III

Supplementary Figure 8: LUAD niche atlas for all tumor samples (magnified version).
UMAP projection of n = 2,233 tumor samples according to niche patterns shows distinct groups (I, II, III; scale bar: 500µm). Each dot on tumor samples corresponds to a single cell, and colors indicate the assigned cellular niche.

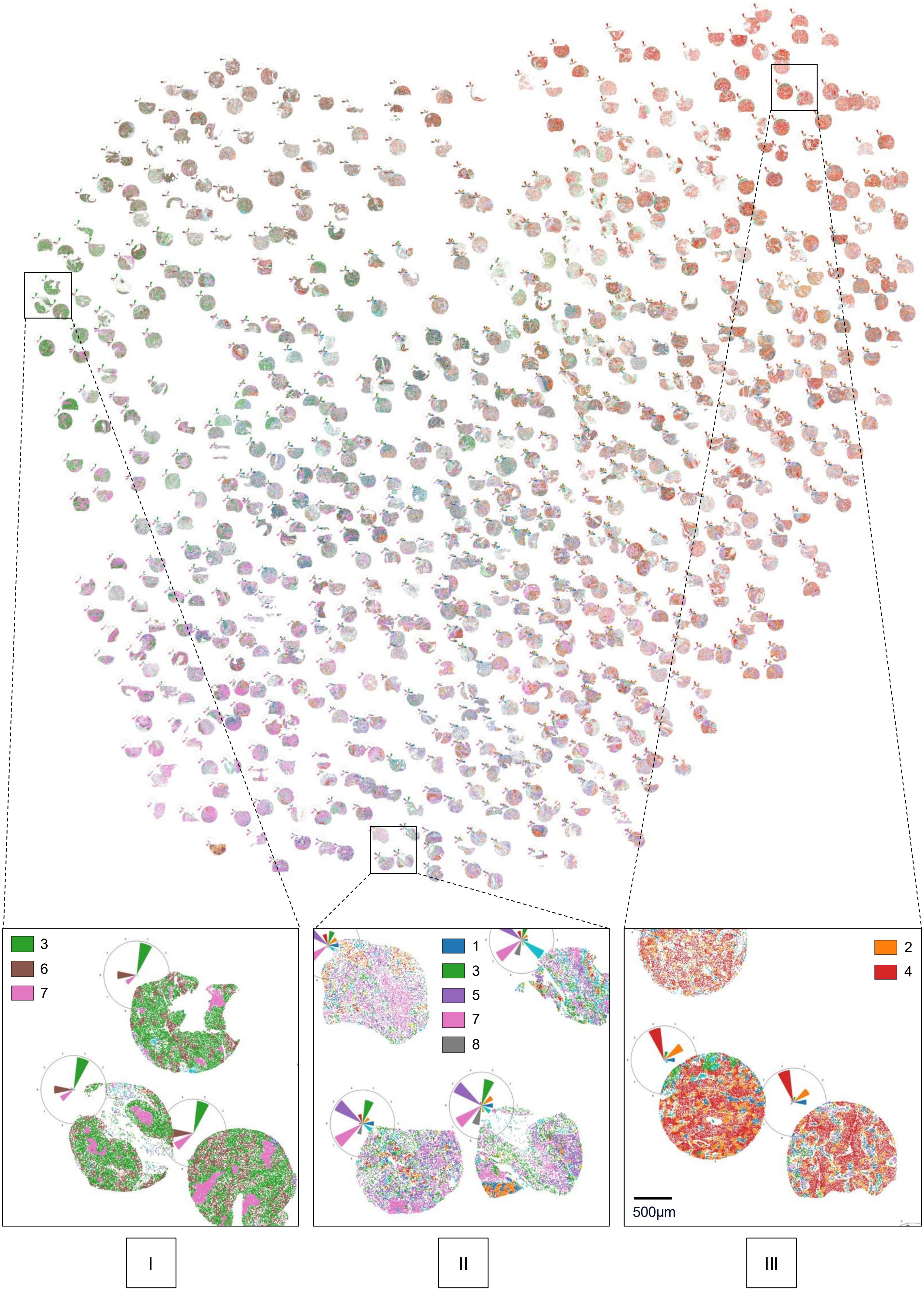
3
6
7
1
3
5
7
8
2
4
500µm
I
II
III

Supplementary Figure 9: LUSC niche atlas for all tumor samples (magnified version).
UMAP projection of n = 1,659 tumor samples according to niche patterns shows distinct groups (I, II, III; scale bar: 500µm). Each dot on tumor samples corresponds to a single cell, and colors indicate the assigned cellular niche.

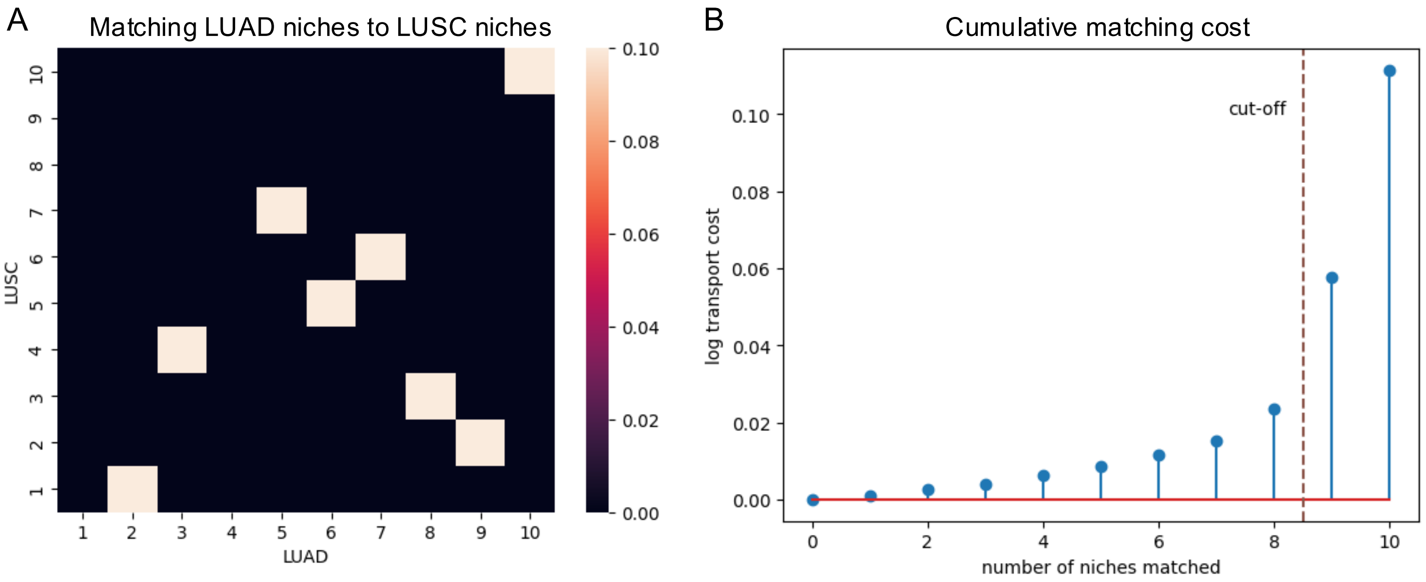

Supplementary Figure 10: Comparison of LUAD and LUSC niches.
(A) We find similarities among the cell niches in LUAD and LUSC in terms of their distribution of contained phenotypes. To suggest a 1:1 matching, we adapt the partial Wasserstein optimal transport formulation with a chi-square-metric and present the solution for an 80% match. (B) We stop the matching process at 80% to avoid excessively high transport costs that appear on the 9th and 10th matches.

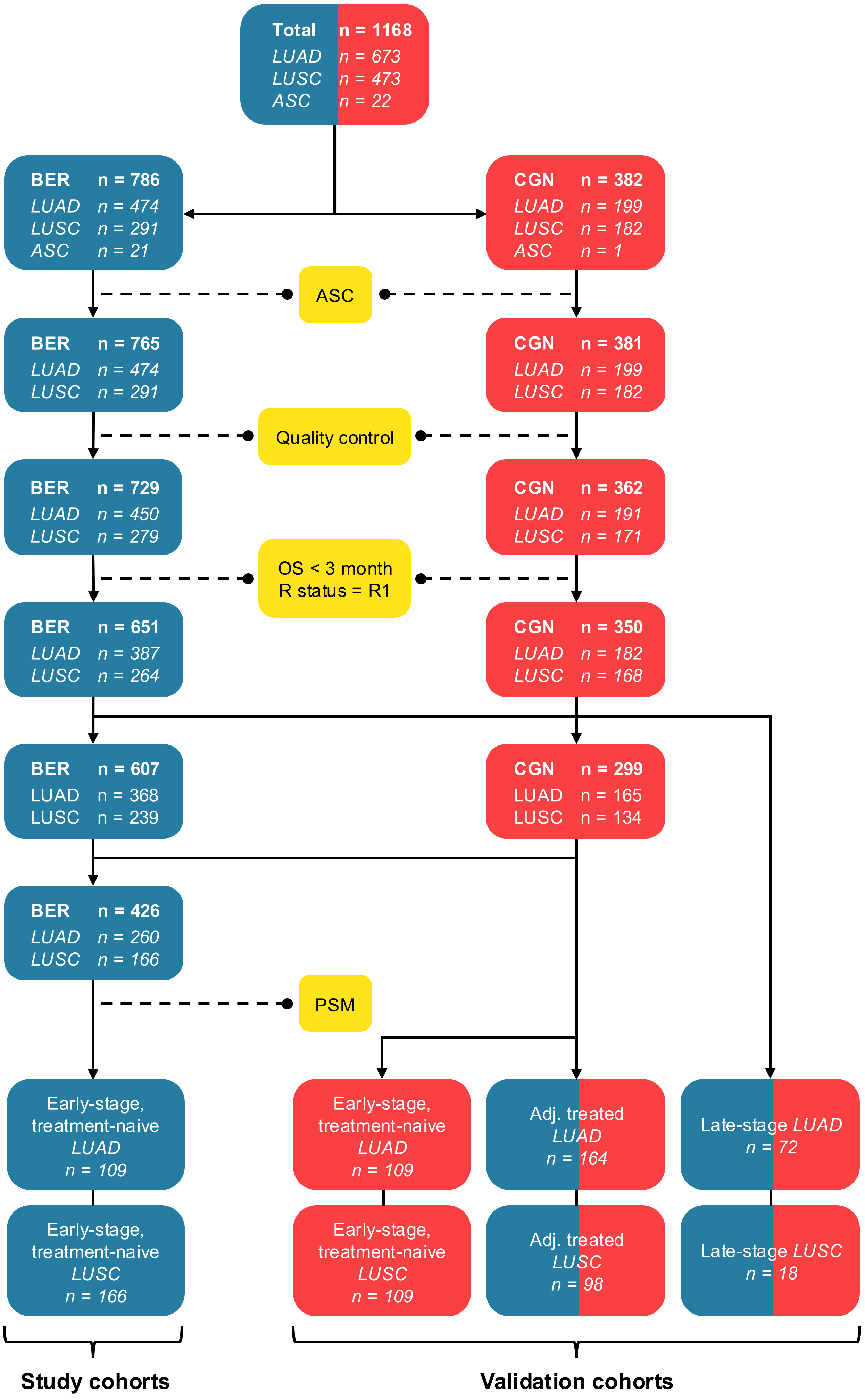
Total n = 1168
LUAD n = 673
LUSC n = 473
ASC n = 22
BER n = 786
LUAD n = 474
LUSC n = 291
ASC n = 21
CGN n = 382
LUAD n = 199
LUSC n = 182
ASC n = 1
ASC
BER n = 765
LUAD n = 474
LUSC n = 291
CGN n = 381
LUAD n = 199
LUSC n = 182
Quality control
BER n = 729
LUAD n = 450
LUSC n = 279
CGN n = 362
LUAD n = 191
LUSC n = 171
OS < 3 month
R status = R1
BER n = 651
LUAD n = 387
LUSC n = 264
CGN n = 350
LUAD n = 182
LUSC n = 168
BER n = 607
LUAD n = 368
LUSC n = 239
CGN n = 299
LUAD n = 165
LUSC n = 134
BER n = 426
LUAD n = 260
LUSC n = 166
PSM
Early-stage, treatment-naive LUAD n = 109
Early-stage, treatment-naive LUSC n = 166
Early-stage, treatment-naive LUAD n = 109
Early-stage, treatment-naive LUSC n = 109
Adj. treated LUAD n = 164
Adj. treated LUSC n = 98
Late-stage LUAD n = 72
Late-stage LUSC n = 18
Study cohorts
Validation cohorts

Supplementary Figure 11: Detailed patient selection flowchart.
The patients from Berlin and Cologne are indicated by blue and red, respectively, while exclusion criteria are highlighted in yellow. ASC (adenosquamous carcinoma), OS (overall survival), R status (resection status), R1 (non in-sano resection), PSM (propensity score matching).

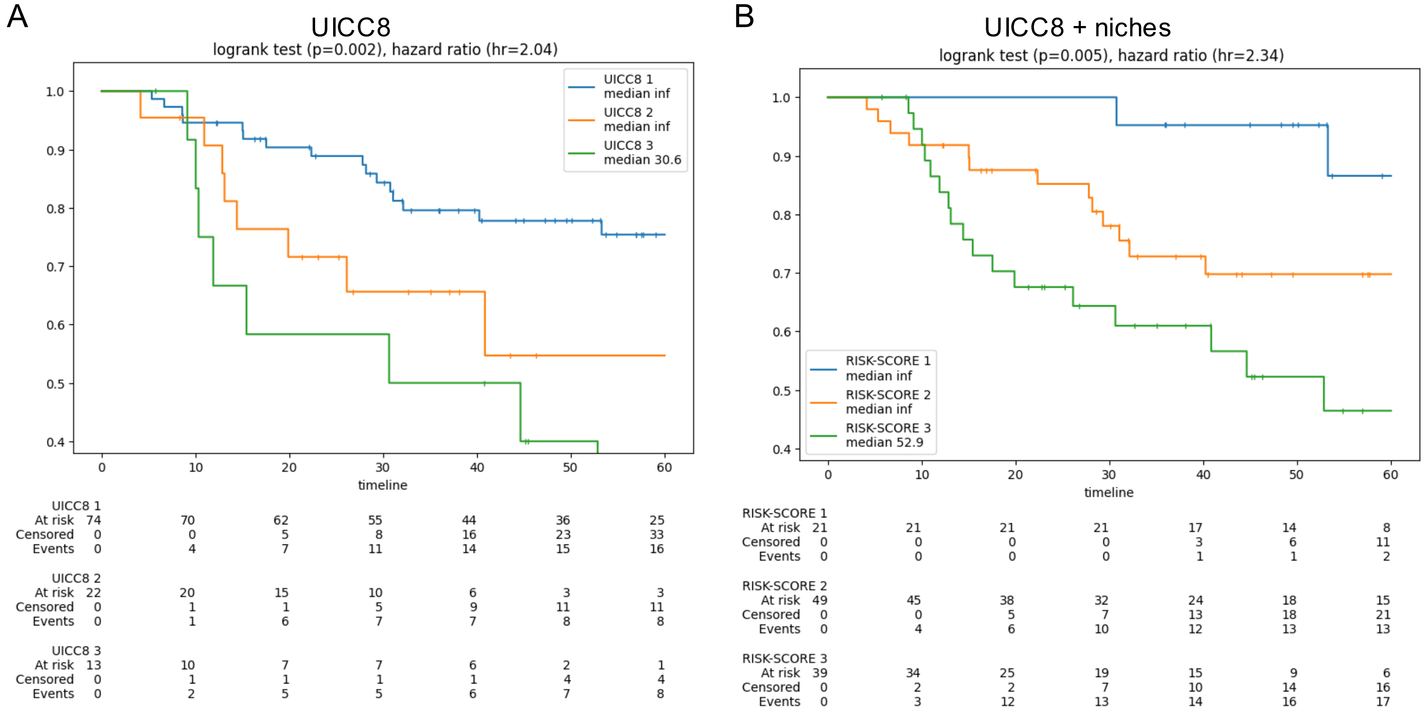

Supplementary Figure 12: Comparison of UICC8 against niches in LUAD.
(A) Kaplan-Meier analysis, patients (n = 109) stratified by the UICC8 stages I-III.
(B) Kaplan-Meier analysis, patients (n = 109) stratified by the risk scores as assigned via the cell-niche survival model. Patients are split into tertiles for better comparison to UICC8.
Separation tested significantly via log-rank test.

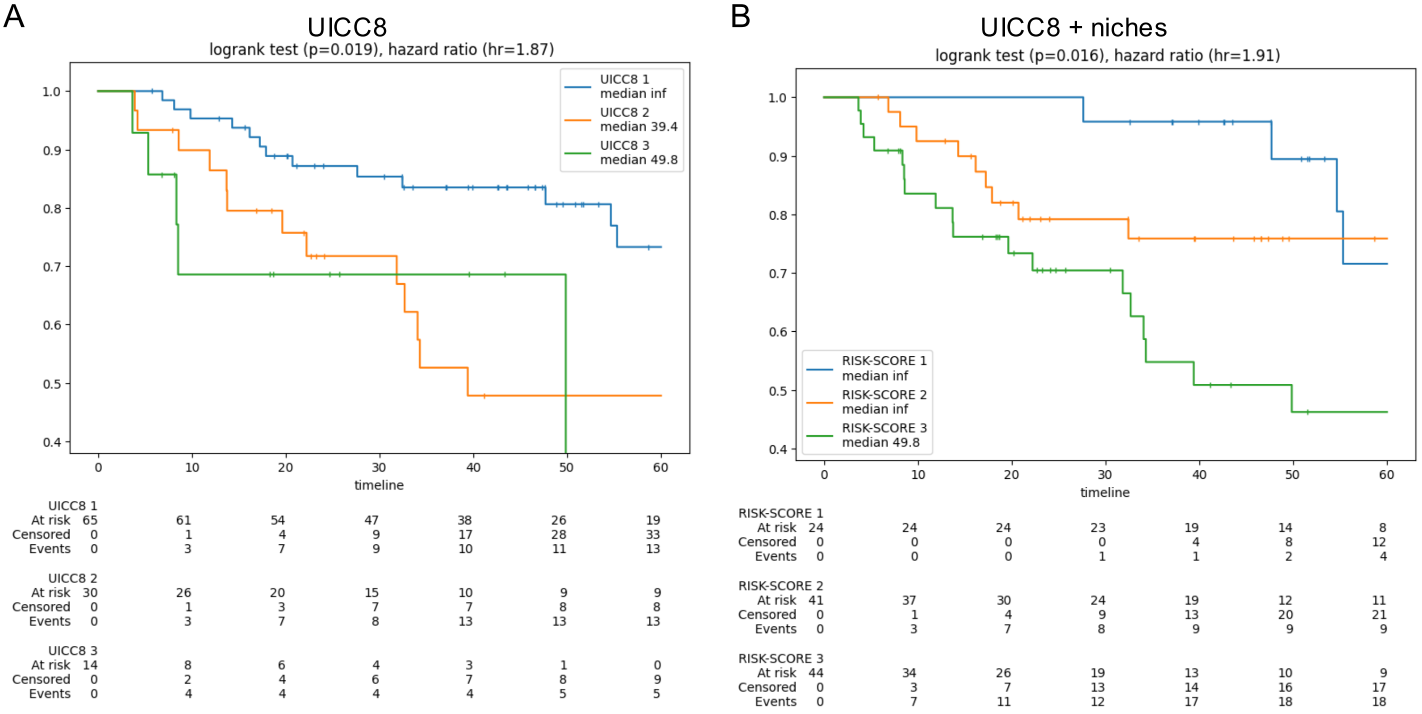

Supplementary Figure 13: Comparison of UICC8 against niches in LUSC.
(A) Kaplan-Meier analysis, patients (n = 109) stratified by the UICC8 stages I-III.
(B) Kaplan-Meier analysis, patients (n = 109) stratified by the risk scores as assigned via the cell-niche survival model. Patients are split into tertiles for better comparison to UICC8**.**
Separation tested significantly via log-rank test.

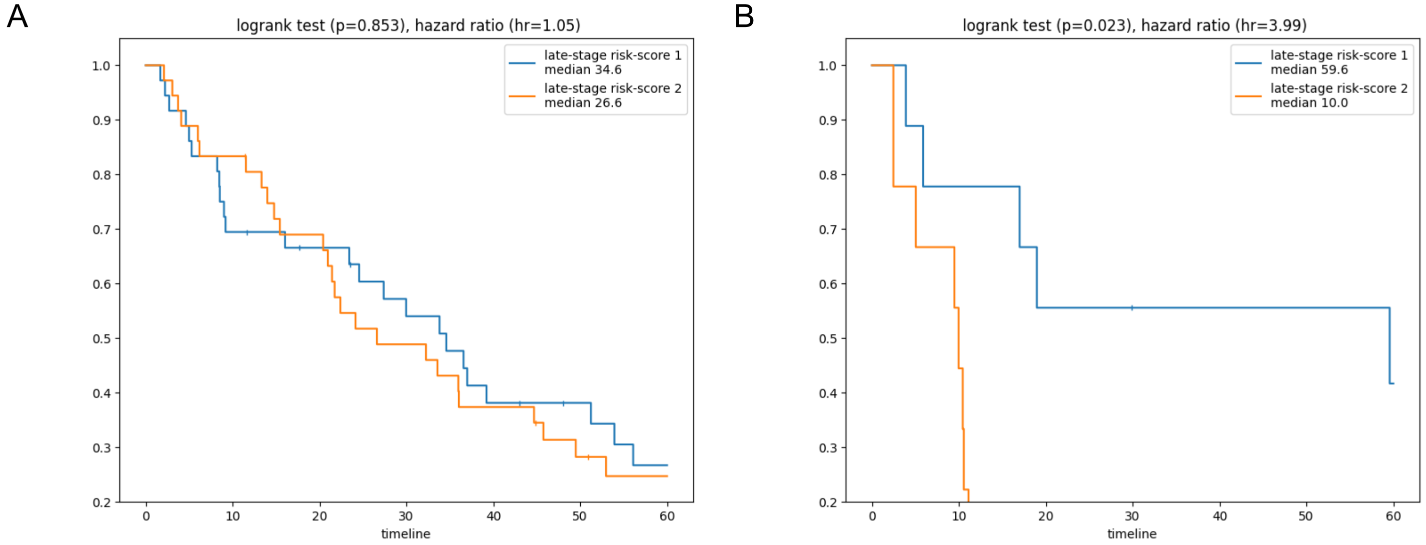

A

late-stage risk-score 1							
At risk	36	25	22	17	12	10	7
Censored	0	0	2	3	3	5	5
Events	0	11	12	16	21	21	24
late-stage risk-score 2							
At risk	36	30	24	17	13	9	7
Censored	0	0	1	1	1	2	3
Events	0	6	11	18	22	25	26

B

late-stage risk-score 1							
At risk	9	7	5	4	4	4	3
Censored	0	0	0	1	1	1	1
Events	0	2	4	4	4	4	5
late-stage risk-score 2							
At risk	9	4	1	1	1	0	0
Censored	0	0	0	0	0	1	1
Events	0	5	8	8	8	8	8

Supplementary Figure 14: Late-stage patients.
(A) LUAD late-stage patients UICC8=IV (n = 72).
(B) LUSC late-stage patients UICC8=IV (n = 18).
Separation tested significantly via log-rank test.

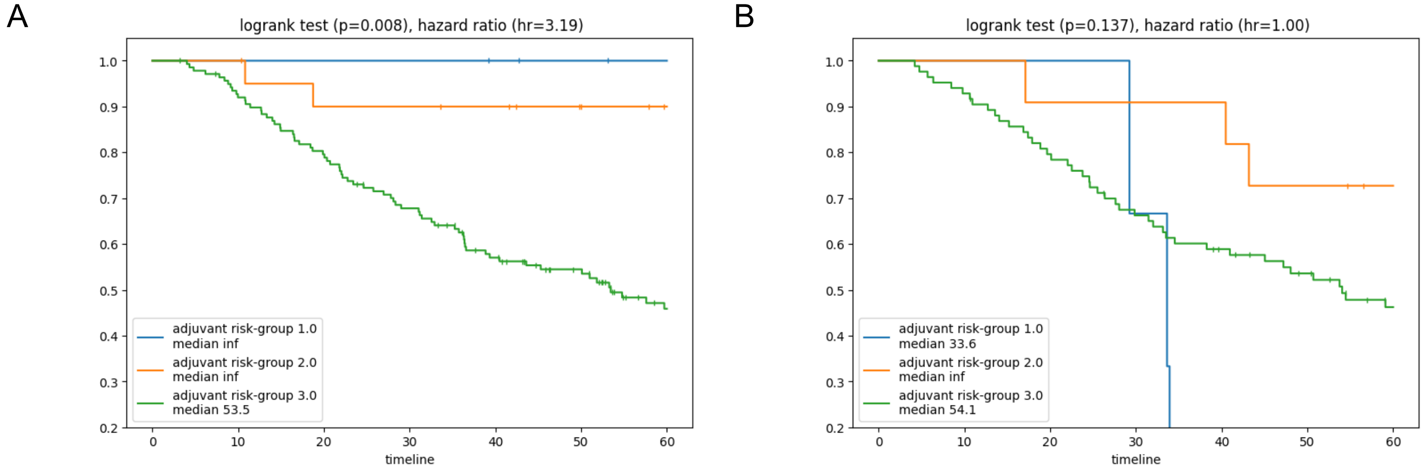

A

	0	10	20	30	40	50	60
**adjuvant risk-group 1.0**							
At risk	4	4	4	4	3	2	1
Censored	0	0	0	0	1	2	3
Events	0	0	0	0	0	0	0
**adjuvant risk-group 2.0**							
At risk	21	21	18	18	17	14	11
Censored	0	0	1	1	2	5	8
Events	0	0	2	2	2	2	2
**adjuvant risk-group 3.0**							
At risk	139	126	109	91	72	57	37
Censored	0	2	2	4	9	21	33
Events	0	11	28	44	58	61	69

B

	0	10	20	30	40	50	60
**adjuvant risk-group 1.0**							
At risk	3	3	3	2	0	0	0
Censored	0	0	0	0	0	0	0
Events	0	0	0	1	3	3	3
**adjuvant risk-group 2.0**							
At risk	11	11	10	10	10	8	6
Censored	0	0	0	0	0	0	2
Events	0	0	1	1	1	3	3
**adjuvant risk-group 3.0**							
At risk	84	78	66	54	46	39	29
Censored	0	0	1	2	4	7	12
Events	0	6	17	28	34	38	43

Supplementary Figure 15: Adjuvant patients.
(A) LUAD adjuvant patients (n = 164).
(B) LUSC adjuvant patients (n = 98).
Separation tested significantly via log-rank test.

A

Gene	*BRAF*	*CTNNB1*	*DDR2*	*EGFR*	*ERBB2*	*FGFR1*	*FGFR2*	*FGFR3*	*IDH1*	*KEAP1*	*KRAS*	*MAP2K1*	*MET*	*NRAS*	*PIK3CA*	*PTEN*	*STK11*	*TP53*
**Exon**	11, 15	3	3-18	18-21	19,20	4-7, 10, 12-15	8-10, 12, 17, 20	7, 10, 15	4	2-6	2,3	2	14	2, 3	10, 21	1-8	2-7	5-8

B

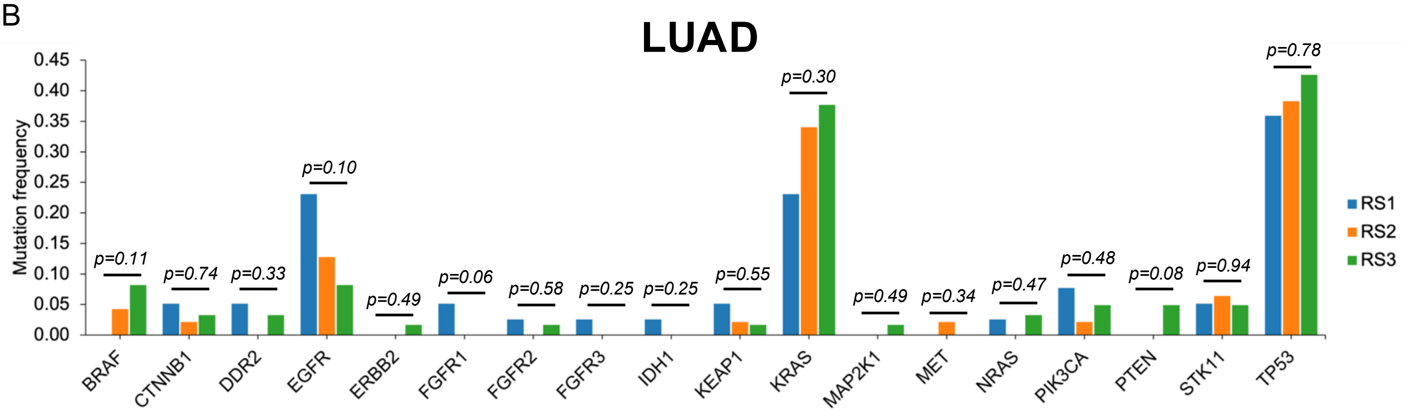

C

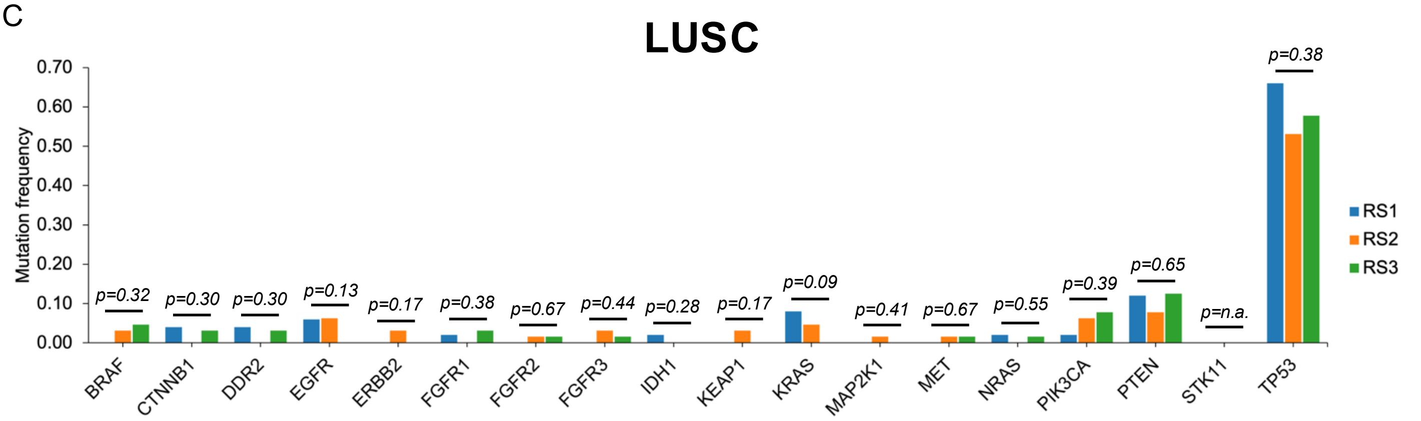

Supplementary Figure 16: Independence of cell-niche derived risk groups and mutation status.
(A) Gene panel overview, including 18 essential genes for the molecular characterization of NSCLC.
(B) Mutation frequencies across cell-niche-derived risk groups in LUAD tested for association using Chi-squared test (n = 147 patients).
(C) Mutation frequencies across cell-niche-derived risk groups in LUSC tested for association using Chi-squared test (n = 178 patients).

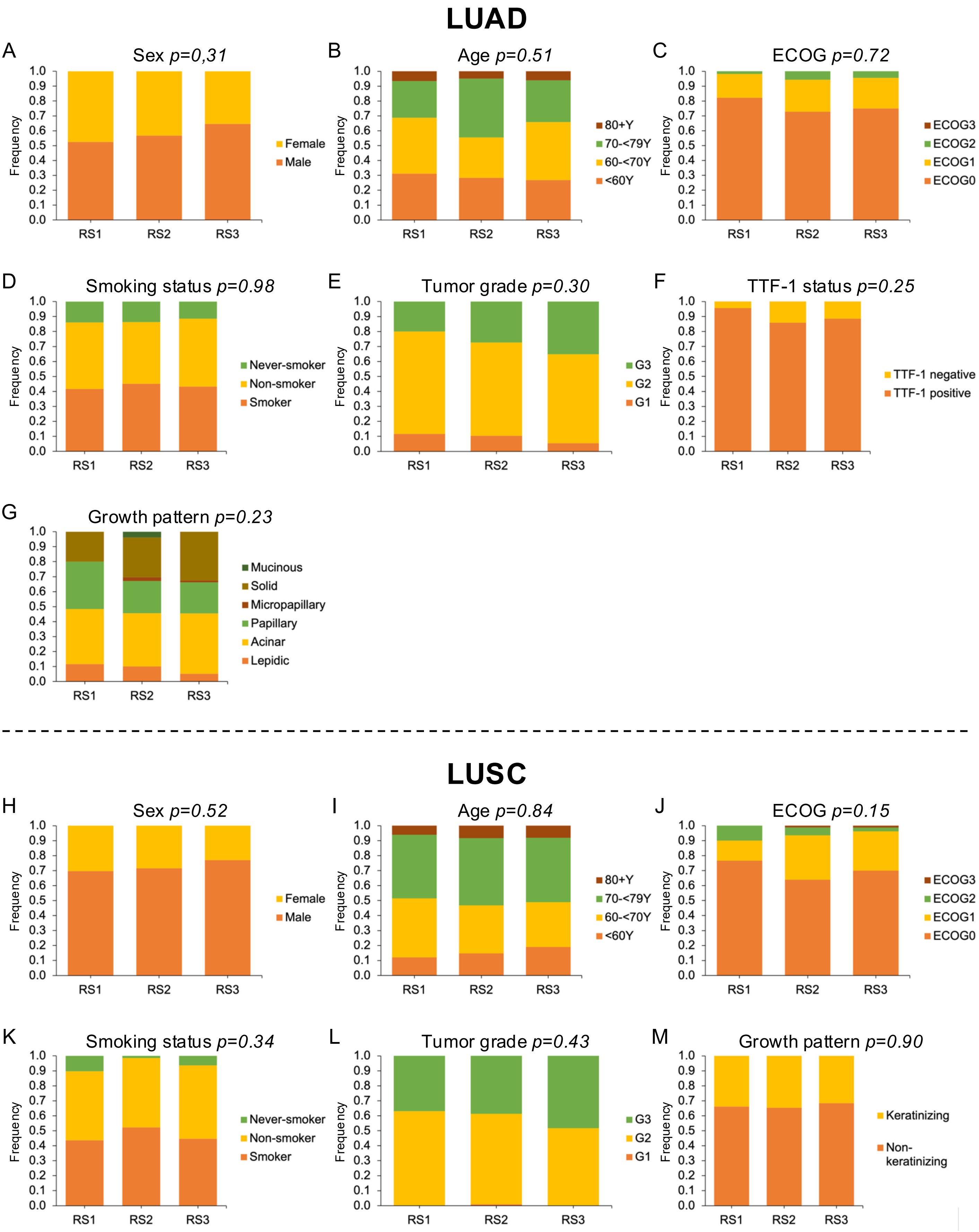
LUAD
A Sex p=0,31
Female
Male
B Age p=0.51
80+Y
70-<79Y
60-<70Y
<60Y
C ECOG p=0.72
ECOG3
ECOG2
ECOG1
ECOG0
D Smoking status p=0.98
Never-smoker
Non-smoker
Smoker
E Tumor grade p=0.30
G3
G2
G1
F TTF-1 status p=0.25
TTF-1 negative
TTF-1 positive
G Growth pattern p=0.23
Mucinous
Solid
Micropapillary
Papillary
Acinar
Lepidic
LUSC
H Sex p=0.52
Female
Male
I Age p=0.84
80+Y
70-<79Y
60-<70Y
<60Y
J ECOG p=0.15
ECOG3
ECOG2
ECOG1
ECOG0
K Smoking status p=0.34
Never-smoker
Non-smoker
Smoker
L Tumor grade p=0.43
G3
G2
G1
M Growth pattern p=0.90
Keratinizing
Non-keratinizing
Frequency
RS1
RS2
RS3
0.0 0.1 0.2 0.3 0.4 0.5 0.6 0.7 0.8 0.9 1.0

Supplementary Figure 17: Cell-niche derived risk scores are independent of established clinicopathological covariates.
(A-G) Distribution of sex (n = 224 patients), age (n = 224 patients), ECOG performance status (n = 194 patients), smoking status (n = 131 patients), tumor grade (n = 211 patients), TTF-1 expression (n = 180 patients), and growth pattern (n = 216 patients) across cell-niche-derived risk groups in LUAD tested for association using Chi-squared test.
(H-M) Distribution of sex (n = 275 patients), age (n = 275 patients), ECOG performance status (n = 234 patients), smoking status (n = 156 patients), tumor grade (n = 256 patients), and growth pattern (n = 270 patients) across cell-niche-derived risk groups in LUSC tested for association using Chi-squared test.

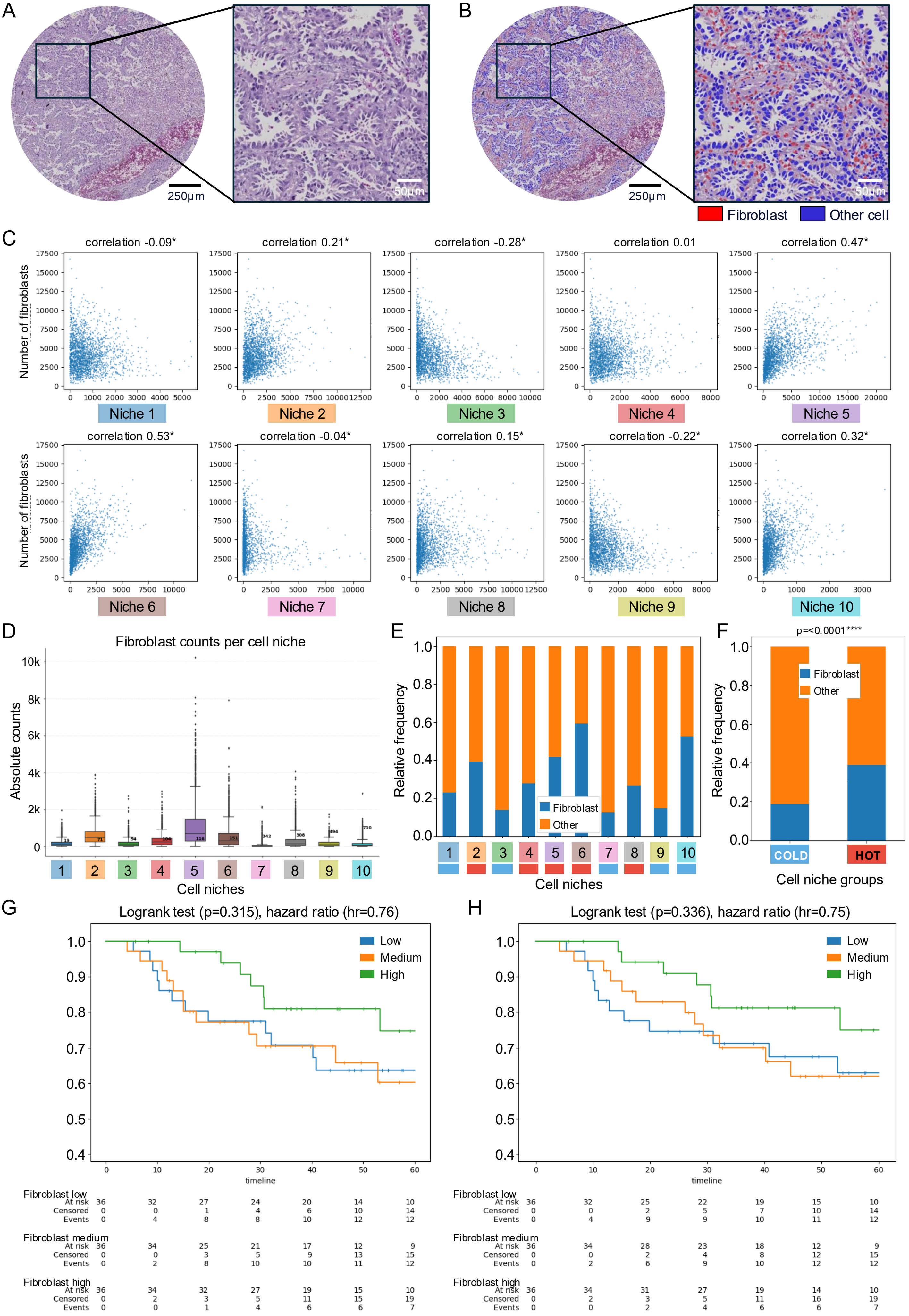

A
250µm
50µm
B
250µm
50µm
Fibroblast
Other cell
C
correlation -0.09*
correlation 0.21*
correlation -0.28*
correlation 0.01
correlation 0.47*
Number of fibroblasts
Niche 1
Niche 2
Niche 3
Niche 4
Niche 5
correlation 0.53*
correlation -0.04*
correlation 0.15*
correlation -0.22*
correlation 0.32*
Niche 6
Niche 7
Niche 8
Niche 9
Niche 10
D
Fibroblast counts per cell niche
Absolute counts
Cell niches
E
Relative frequency
Fibroblast
Other
Cell niches
F
p=<0.0001****
Relative frequency
Fibroblast
Other
COLD
HOT
Cell niche groups
G
Logrank test (p=0.315), hazard ratio (hr=0.76)
Low
Medium
High
timeline
Fibroblast low
At risk 36 32 27 24 20 14 10
Censored 0 0 1 4 6 10 14
Events 0 4 8 8 10 12 12
Fibroblast medium
At risk 36 34 25 21 17 12 9
Censored 0 0 3 5 9 13 15
Events 0 2 8 10 10 11 12
Fibroblast high
At risk 36 34 32 27 19 15 10
Censored 0 2 3 5 11 15 19
Events 0 0 1 4 6 6 7
H
Logrank test (p=0.336), hazard ratio (hr=0.75)
Low
Medium
High
timeline
Fibroblast low
At risk 36 32 25 22 19 15 10
Censored 0 0 2 5 7 10 14
Events 0 4 9 9 10 11 12
Fibroblast medium
At risk 36 34 28 23 18 12 9
Censored 0 0 2 4 8 12 15
Events 0 2 6 9 10 12 12
Fibroblast high
At risk 36 34 31 27 19 14 10
Censored 0 2 3 5 11 16 19
Events 0 0 2 4 6 6 7

Supplementary Figure 18: Fibroblast abundance across cell niches in LUAD.
(A) Representative H&E image of a tissue spot (n = 1) containing LUAD (left) and corresponding highmagnification zoom-in (right).
(B) Output of the cell classification model showing AI-derived fibroblast predictions (red polygons) and other cell predictions (blue polygons) for the same spot (n = 1) and corresponding zoomed region shown in (A).
(C) Correlation of fibroblast abundance with niche abundance across tissue spots (n = 2,189 from n = 663 patients); each dot represents one tissue spot. Statistical significance was assessed using Pearson's correlation (scipy.stats.pearsonr).
(D) Absolute fibroblast counts per cell niche (n = 663 patients).
(E) Relative fibroblast frequency per cell niche, with niches classified as hot (red bar at bottom) or cold (blue bar at bottom; n = 663 patients).
(F) Relative fibroblast frequency within cold (left) and hot (right) niche groups (n = 663 patients). Statistical significance was assessed using a Chi-squared test.
(G) Kaplan-Meier analysis stratified by fibroblast maximum abundance over tissue spots per patient (max pooling); patients (n = 108) were divided into tertiles for direct comparison with cell niche pattern-based risk scores. Separation tested significantly via log-rank test.
(H) Kaplan–Meier analysis stratified by fibroblast mean abundance over tissue spots per patient (n = 108); patients were divided into tertiles for direct comparison with cell niche pattern-based risk scores. Separation tested significantly via log-rank test.
Scale bars: (A+B) 250μm (tissue spots) and 50μm (magnified regions).

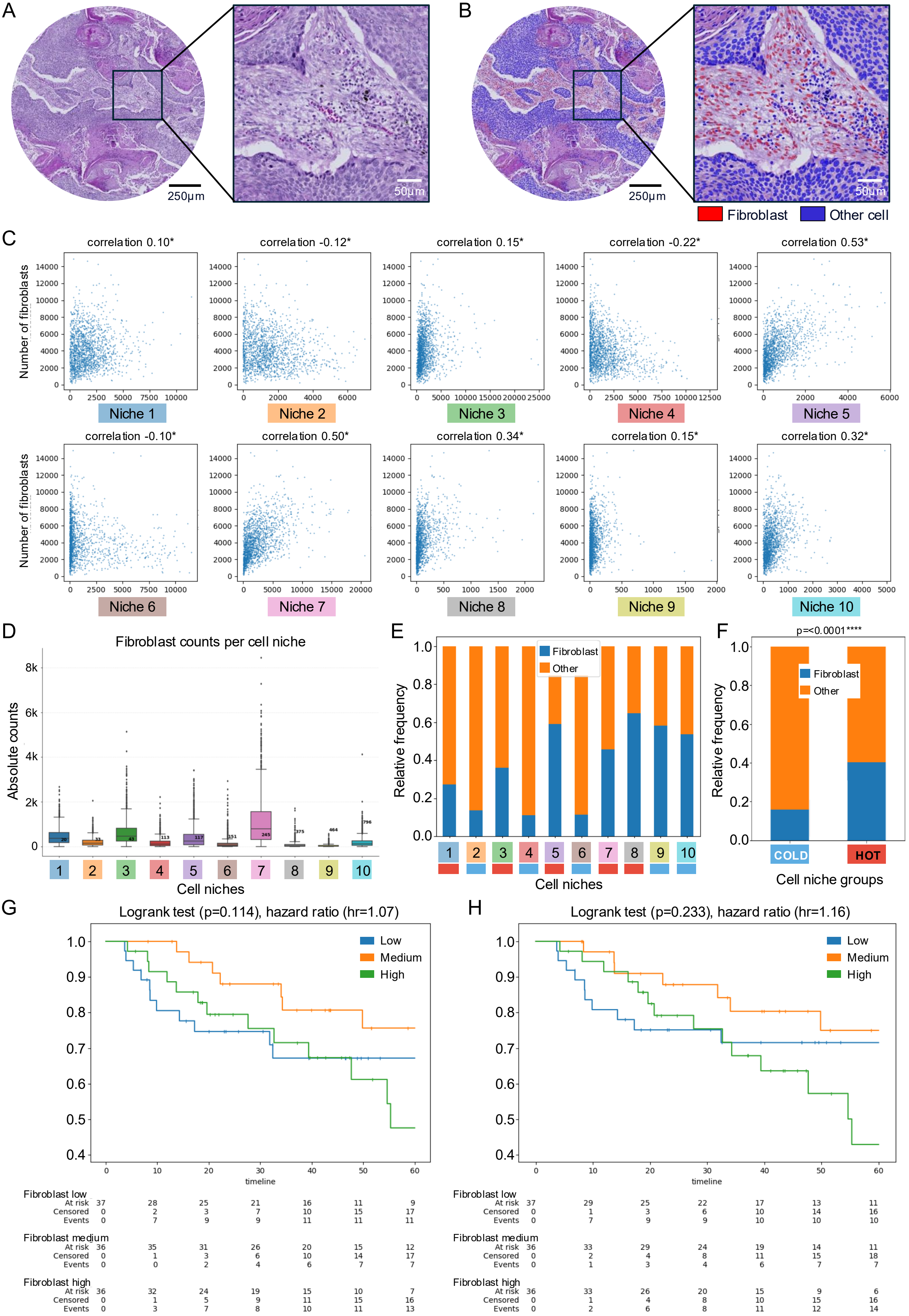
A
250μm
50μm
B
250μm
50μm
Fibroblast
Other cell
C
correlation 0.10*
correlation -0.12*
correlation 0.15*
correlation -0.22*
correlation 0.53*
Number of fibroblasts
Niche 1
Niche 2
Niche 3
Niche 4
Niche 5
correlation -0.10*
correlation 0.50*
correlation 0.34*
correlation 0.15*
correlation 0.32*
Niche 6
Niche 7
Niche 8
Niche 9
Niche 10
D
Fibroblast counts per cell niche
Absolute counts
Cell niches
E
Fibroblast
Other
Relative frequency
Cell niches
F
p=<0.0001****
Fibroblast
Other
Relative frequency
COLD
HOT
Cell niche groups
G
Logrank test (p=0.114), hazard ratio (hr=1.07)
Low
Medium
High
timeline
Fibroblast low
At risk 37 28 25 21 16 11 9
Censored 0 2 3 7 10 15 17
Events 0 7 9 9 11 11 11
Fibroblast medium
At risk 36 35 31 26 20 15 12
Censored 0 1 3 6 10 14 17
Events 0 0 2 4 6 7 7
Fibroblast high
At risk 36 32 24 19 15 10 7
Censored 0 1 5 9 11 15 16
Events 0 3 7 8 10 11 13
H
Logrank test (p=0.233), hazard ratio (hr=1.16)
Low
Medium
High
timeline
Fibroblast low
At risk 37 29 25 22 17 13 11
Censored 0 1 3 6 10 14 16
Events 0 7 9 9 10 10 10
Fibroblast medium
At risk 36 33 29 24 19 14 11
Censored 0 2 4 8 11 15 18
Events 0 1 3 4 6 7 7
Fibroblast high
At risk 36 33 26 20 15 9 6
Censored 0 1 4 8 10 15 16
Events 0 2 6 8 11 12 14

Supplementary Figure 19: Fibroblast abundance across cell niches in LUSC.
(A) Representative H&E image of a tissue spot (n = 1) containing LUSC (left) and corresponding high magnification zoom-in (right).
(B) Output of the cell classification model showing AI-derived fibroblast predictions (red polygons) and other cell predictions (blue polygons) for the same spot (n = 1) and corresponding zoomed region shown in (A).
(C) Correlation of fibroblast abundance with niche abundance across tissue spots (n = 1,657 from n = 462 patients); each dot represents one tissue spot. Statistical significance was assessed using Pearson's correlation (scipy.stats.pearsonr).
(D) Absolute fibroblast counts per cell niche (n = 462 patients).
(E) Relative fibroblast frequency per cell niche, with niches classified as hot (red bar at bottom) or cold (blue bar at bottom; n = 462 patients).
(F) Relative fibroblast frequency within cold (left) and hot (right) niche groups (n = 462 patients). Statistical significance was assessed using a Chi-squared test.
(G) Kaplan-Meier analysis stratified by fibroblast maximum abundance over tissue spots per patient (max Pooling); patients (n = 108) were divided into tertiles for direct comparison with cell niche pattern-based risk scores. Separation tested significantly via log-rank test.
(H) Kaplan–Meier analysis stratified by fibroblast mean abundance over tissue spots per patient (n = 109); patients were divided into tertiles for direct comparison with cell niche pattern-based risk scores. Separation tested significantly via log-rank test.
Scale bars: (A+B) 250µm (tissue spots) and 50µm (magnified regions).

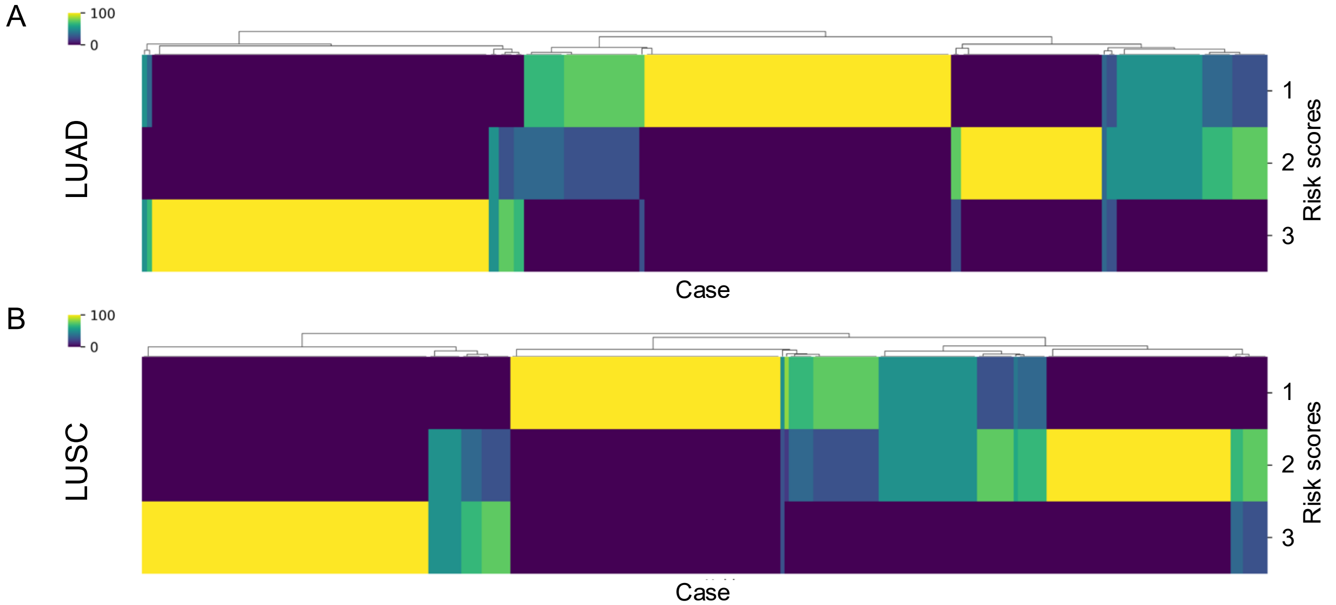

C

Core distribution by risk group	LUAD	LUSC
Patients with all 4 cores in the same risk group	70%	65%
Patients with ≥ 75% of cores in the same risk group	75%	71%
Patients with ≥ 50% of cores in the same risk group	87%	87%

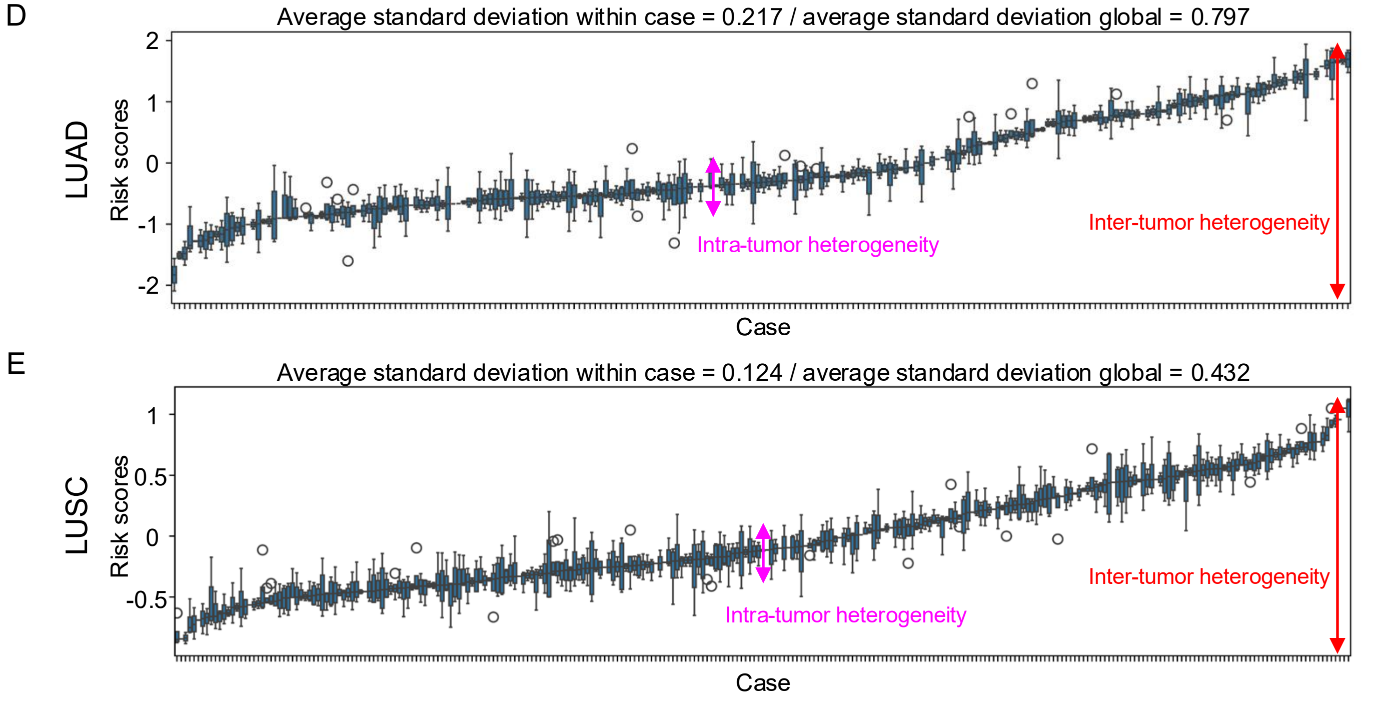

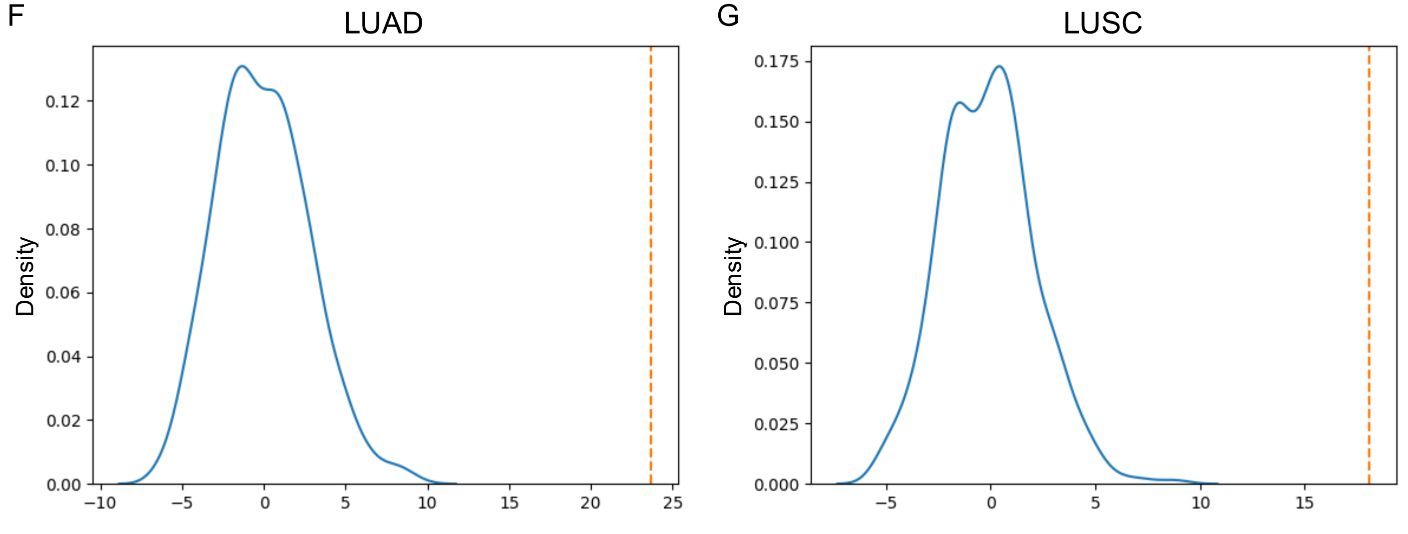

Supplementary Figure 20: Comprehensive analysis of inter- and intra-tumor heterogeneity.
(A) LUAD: Cluster map illustrates the distribution of tissue spots (n = 2,233 from n = 663 patients), which have been assigned one of three risk scores. Yellow indicates cases where all tissue cores share the same risk score. Conversely, dark blue is used to indicate that no cores of one risk score are observed in a case. Green and blue represent cases with
mixed scores. y-axis: risk score, x-axis: cases.
(B) Same as (A) but for LUSC (n = 1,659 tissue spots from n = 462 patients).
(C) Tissue core distribution by risk scores for LUAD (n = 663 patients) and LUSC (n = 462 patients). The results show a relatively high concordance/low heterogeneity with 70% (65%) of patients showing the same risk group in all 4 TMA cores.
(D) LUAD: Continuous risk score plot shows that intra-tumor heterogeneity is significantly lower than interpatient (inter-tumor) heterogeneity (n = 663 patients). y-axis: risk score, x-axis: cases. Each small box plot corresponds to one patient.
(E) Same as (D) but for LUSC (n = 462 patients).
(F+G) "Within-risk group" niche pattern variation is significantly lower than "between-risk group" variation in LUAD (F; n = 663 patients) and LUSC (G; n = 462 patients):
A permutation test was performed that compares the difference of niche pattern variation within the same risk group and the variation observed across the entire cohort ($p<0.001$****). Blue curve: distribution of results when the risk scores for the entire cohort were randomly shuffled (null hypothesis). Orange line: observed difference in variation (transport costs) using the actual risk groups.

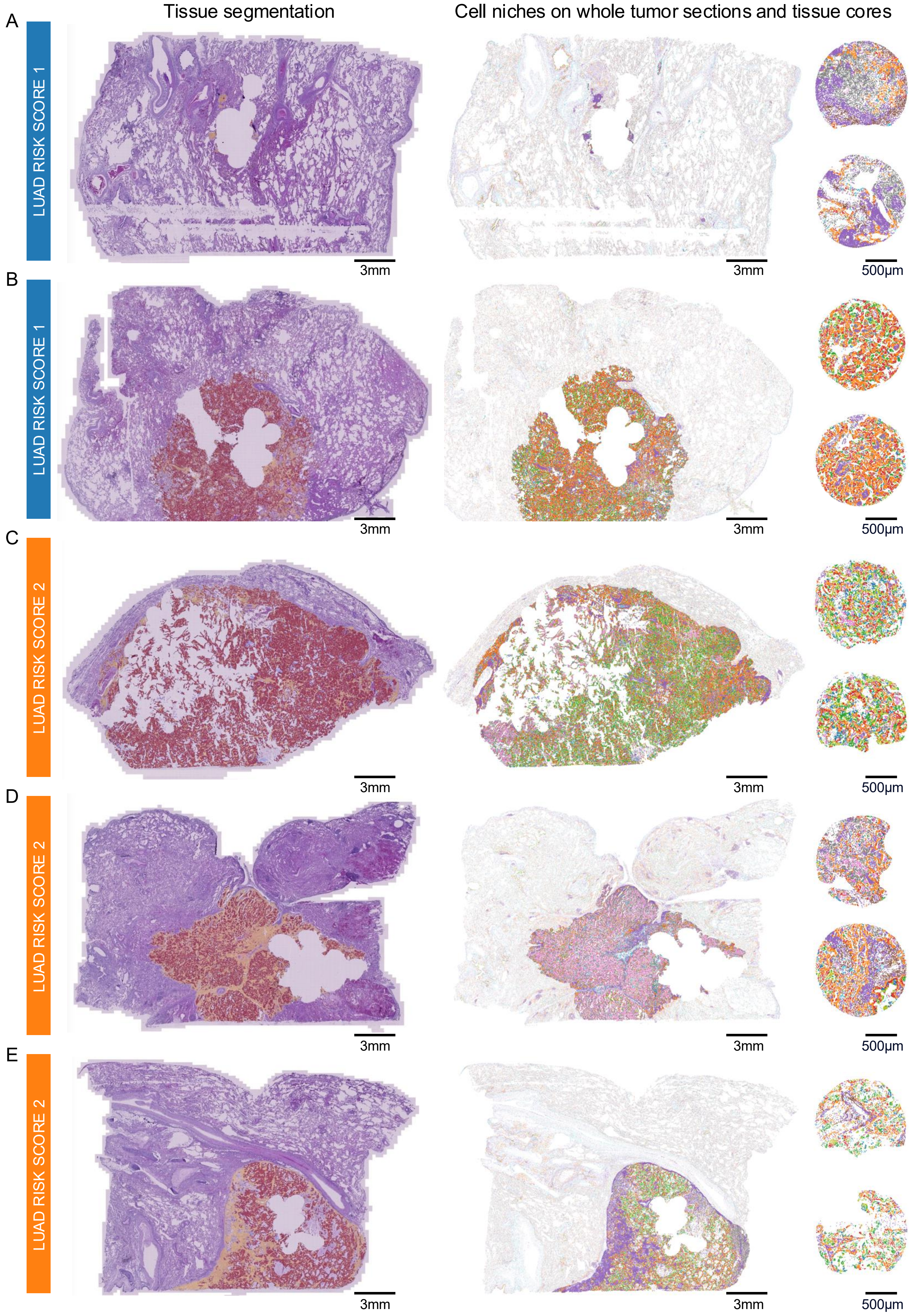

Tissue segmentation
Cell niches on whole tumor sections and tissue cores
A
LUAD RISK SCORE 1
3mm
3mm
500μm
B
LUAD RISK SCORE 1
3mm
3mm
500μm
C
LUAD RISK SCORE 2
3mm
3mm
500μm
D
LUAD RISK SCORE 2
3mm
3mm
500μm
E
LUAD RISK SCORE 2
3mm
3mm
500μm

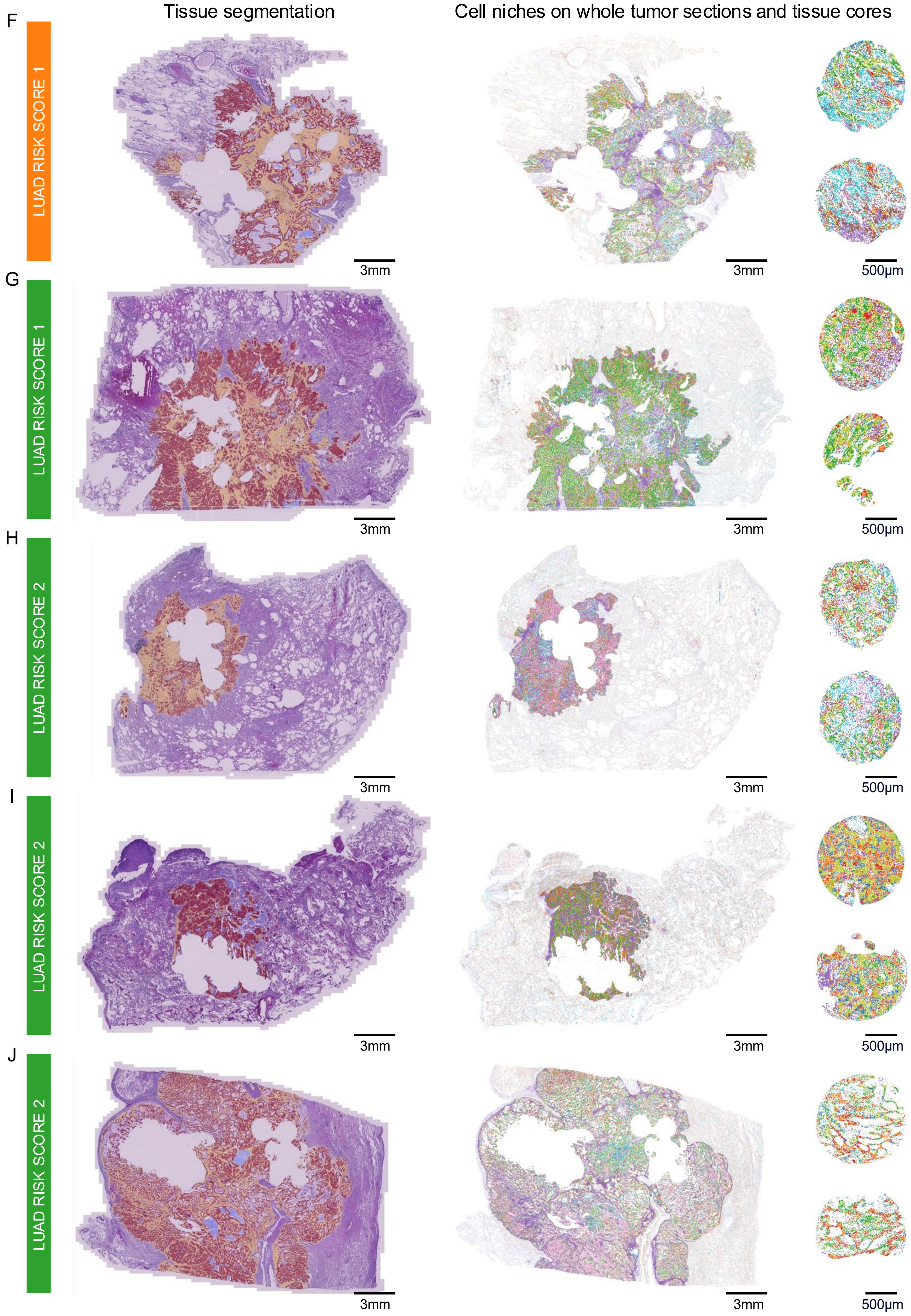
Tissue segmentation
Cell niches on whole tumor sections and tissue cores
F
LUAD RISK SCORE 1
3mm
3mm
500µm
G
LUAD RISK SCORE 1
3mm
3mm
500µm
H
LUAD RISK SCORE 2
3mm
3mm
500µm
I
LUAD RISK SCORE 2
3mm
3mm
500µm
J
LUAD RISK SCORE 2
3mm
3mm
500µm

Supplementary Figure 21: Niche pattern on whole tumor sections and corresponding tissue cores in LUAD.

(A+B) H&E-stained whole tumor section (n = 2 tumor samples) with AI-derived tissue segmentation (left; carcinoma = red, stroma = yellow, necrosis = blue, healthy tissue = uncolored), same whole tumor section with niche patterns of risk

score 1 (center) and two corresponding tissue cores with niche patterns of risk score 1 (right).

(C-F) Same as (A) but for risk score 2 (n = 4 tumor samples).

(G-J) Same as (A) but for risk score 3 (n = 4 tumor samples).

Scale bars: (A-J) 3mm (whole tumor sections) and 500µm (tissue spots)

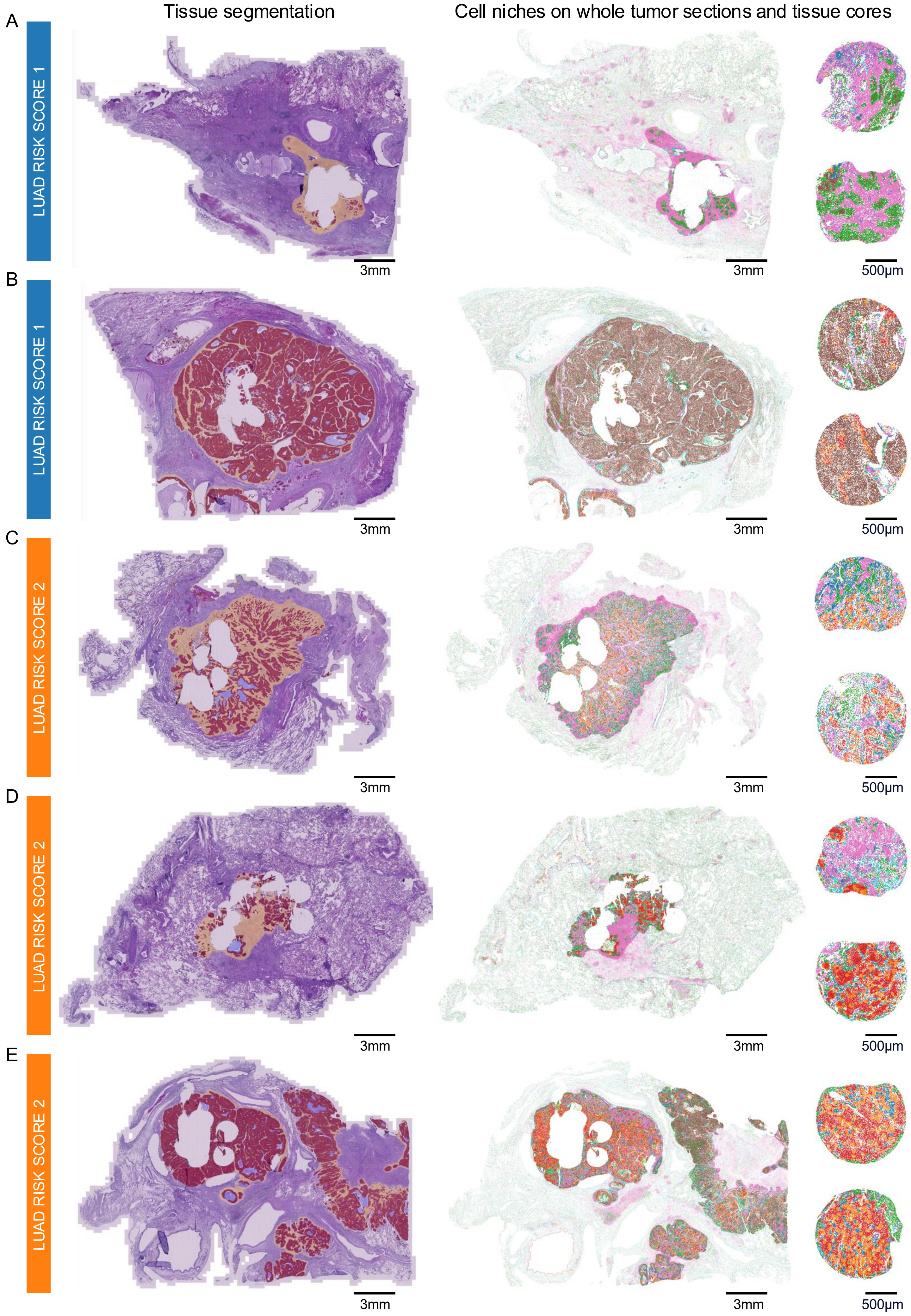

Tissue segmentation
Cell niches on whole tumor sections and tissue cores
A
LUAD RISK SCORE 1
3mm
3mm
500µm
B
LUAD RISK SCORE 1
3mm
3mm
500µm
C
LUAD RISK SCORE 2
3mm
3mm
500µm
D
LUAD RISK SCORE 2
3mm
3mm
500µm
E
LUAD RISK SCORE 2
3mm
3mm
500µm

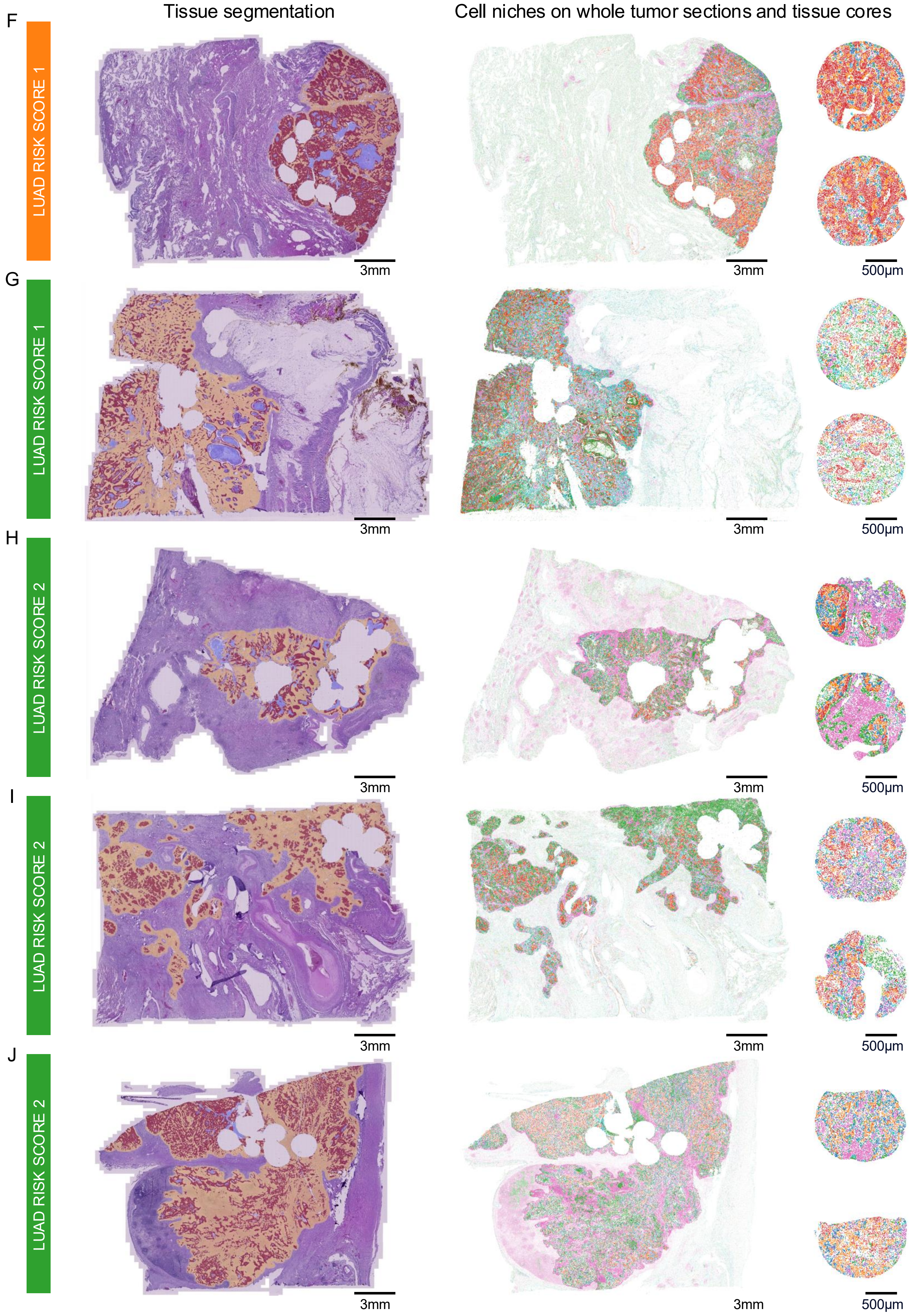
Tissue segmentation
Cell niches on whole tumor sections and tissue cores
F
LUAD RISK SCORE 1
3mm
3mm
500µm
G
LUAD RISK SCORE 1
3mm
3mm
500µm
H
LUAD RISK SCORE 2
3mm
3mm
500µm
I
LUAD RISK SCORE 2
3mm
3mm
500µm
J
LUAD RISK SCORE 2
3mm
3mm
500µm

Supplementary Figure 22: Niche pattern on whole tumor sections and corresponding tissue cores in LUSC.

(A+B) H&E-stained whole tumor section (n = 2 tumor samples) with AI-derived tissue segmentation (left; carcinoma = red, stroma = yellow, necrosis = blue, healthy tissue = uncolored), same whole tumor section with niche patterns of risk

score 1 (center) and two corresponding tissue cores with niche patterns of risk score 1 (right).

(C-F) Same as (A) but for risk score 2 (n = 4 tumor samples).

(G-J) Same as (A) but for risk score 3 (n = 4 tumor samples).

Scale bars: (A-J) 3mm (whole tumor sections) and 500µm (tissue spots)

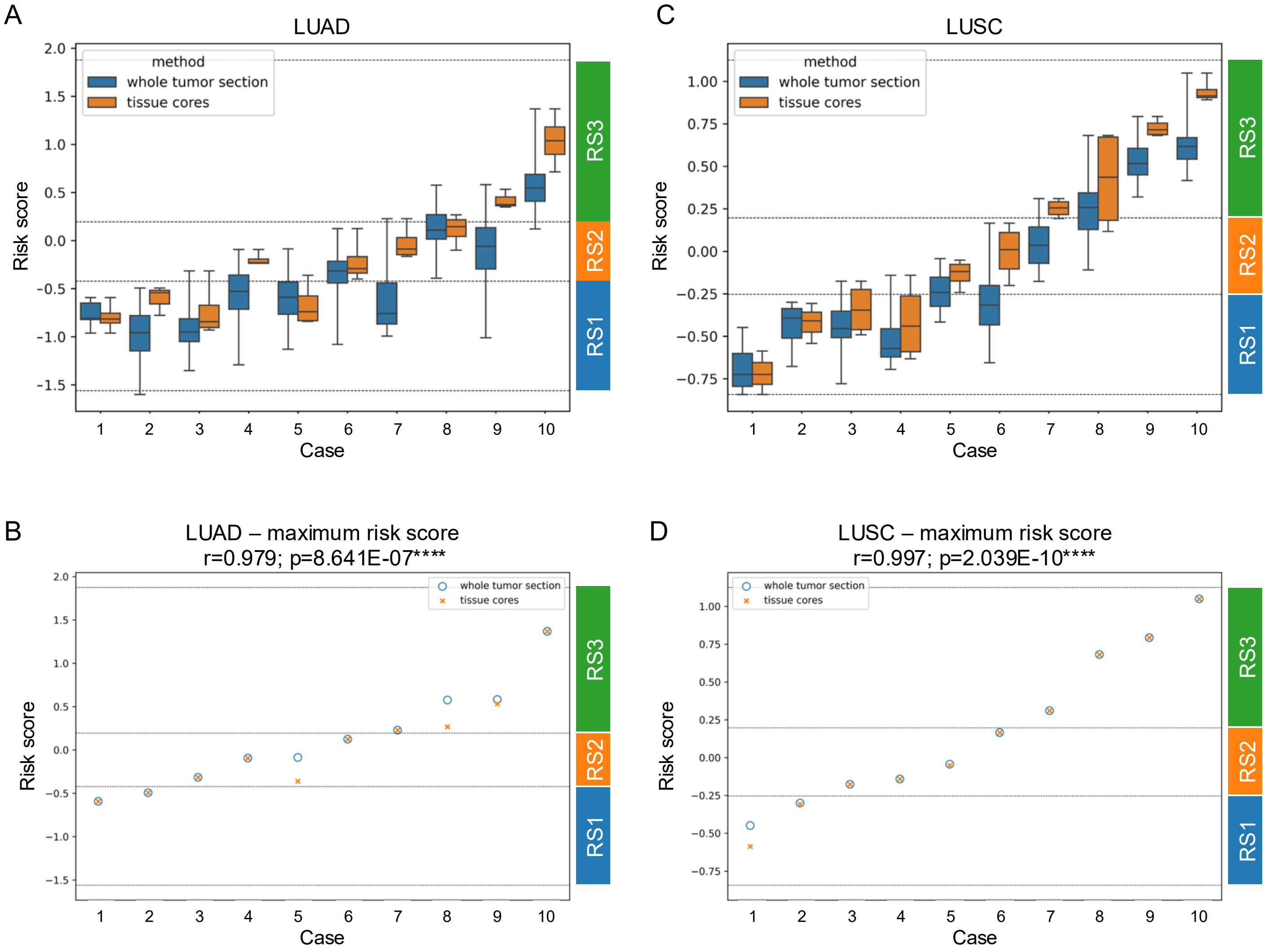

Supplementary Figure 23: Concordance of risk scores between whole tumor sections and corresponding tissue cores.

(A) LUAD risk score distribution (n = 10 patients): A continuous risk score plot shows risk score distributions within the whole tumor sections (blue) and corresponding tissue microarray cores (orange). y-axis: risk score, x-axis: cases (ordered by increasing mean risk), horizontal lines show boundaries between risk scores. Box plots show the median (center line), interquartile range Q1–Q3 (box), and whiskers to the most extreme points within 1.5×IQR.

(B) LUAD risk score concordance (n = 10 patients): Risk scores (maximum risk) for whole tumor sections (blue circles) are highly correlated with tissue core risk scores (orange crosses). Risk group assignments (RS1-3) are 100%

concordant. y-axis: risk score, x-axis: cases (ordered by increasing final risk score), horizontal lines show boundaries between risk scores. Box plots show the median (center line), interquartile range Q1–Q3 (box), and whiskers to the most extreme points within 1.5×IQR.

(C) LUSC risk score distribution (n = 10 patients): Same as (A) but for LUSC. Statistical significance was assessed using Pearson’s correlation (scipy.stats.pearsonr).

(D) LUSC risk score concordance (n = 10 patients): Same as (B) but for LUSC. Statistical significance was assessed using Pearson’s correlation (scipy.stats.pearsonr).

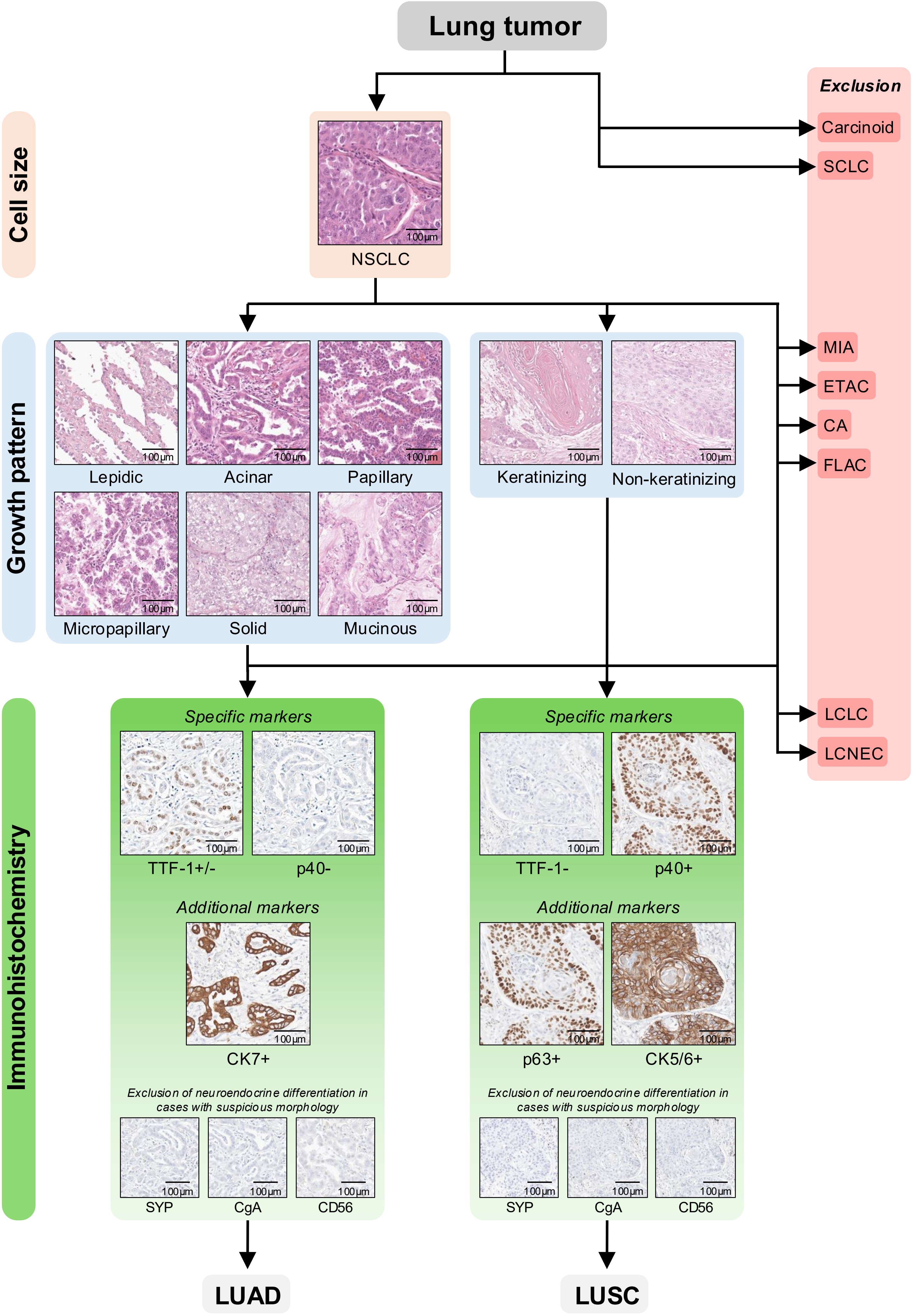

Lung tumor
Cell size
NSCLC
Exclusion
Carcinoid
SCLC
Growth pattern
Lepidic
Acinar
Papillary
Micropapillary
Solid
Mucinous
Keratinizing
Non-keratinizing
MIA
ETAC
CA
FLAC
LCLC
LCNEC
Immunohistochemistry
Specific markers
TTF-1+/-
p40-
Additional markers
CK7+
Exclusion of neuroendocrine differentiation in cases with suspicious morphology
SYP
CgA
CD56
Specific markers
TTF-1-
p40+
Additional markers
p63+
CK5/6+
Exclusion of neuroendocrine differentiation in cases with suspicious morphology
SYP
CgA
CD56
100 µm
LUAD
LUSC

Supplementary Figure 24: Diagnostic workflow and case selection criteria for lung cancer specimens.
Schematic overview of the diagnostic work-up based on histomorphology and immunohistochemistry, including inclusion and exclusion criteria applied for LUAD and LUSC case selection (n = 11 representative tumor samples). Rare tumor types and
neuroendocrine neoplasms were excluded as outlined.
NSCLC, non-small cell lung cancer; SCLC, small-cell lung carcinoma; MIA, minimally invasive adenocarcinoma; ETAC, enteric-type adenocarcinoma; CA, colloid adenocarcinoma; FLAC, fetal adenocarcinoma; LCLC, large-cell lung carcinoma; LCNEC, large-cell neuroendocrine carcinoma; TTF-1, thyroid transcription factor 1; CK, cytokeratin; SYP, Synaptophysin; CgA, Chromogranin A; LUAD, lung adenocarcinoma; LUSC, lung squamous cell carcinoma.
Scale bars: 100µm

	Standard stardist		Standard stardist (DAPI)		In-house (H&E)	
Case number	*recall (sensitivity)*	*precision (PPV)*	*recall (sensitivity)*	*precision (PPV)*	*recall (sensitivity)*	*precision (PPV)*
1	0.496	0.990	0.962	0.830	0.959	0.872
2	0.417	0.981	0.886	0.849	0.941	0.902
3	0.639	0.996	0.923	0.858	0.934	0.919
4	0.509	0.975	0.848	0.800	0.880	0.941
5	0.260	0.990	0.811	0.868	0.904	0.942
6	0.276	0.644	0.916	0.788	0.823	0.933
7	0.667	0.993	0.933	0.838	0.952	0.881
8	0.670	0.974	0.875	0.912	0.923	0.953
9	0.383	0.982	0.952	0.719	0.962	0.921
10	0.537	1.000	0.897	0.846	0.937	0.959
11	0.517	0.896	0.902	0.770	0.919	0.881
12	0.798	0.829	0.920	0.858	0.901	0.908
13	0.594	0.940	0.802	0.867	0.948	0.834
14	0.822	0.899	0.922	0.851	0.897	0.970
15	0.653	0.982	0.955	0.802	0.961	0.955
16	0.394	0.993	0.806	0.848	0.872	0.941
17	0.539	0.978	0.913	0.785	0.827	0.934
18	0.441	0.888	0.810	0.744	0.792	0.867
19	0.496	0.986	0.908	0.826	0.911	0.945
20	0.578	0.952	0.910	0.831	0.902	0.949
21	0.311	0.974	0.836	0.751	0.845	0.953
22	0.193	0.980	0.926	0.765	0.897	0.874
23	0.727	0.959	0.917	0.867	0.919	0.954
24	0.734	0.969	0.789	0.907	0.902	0.946
25	0.436	1.000	0.923	0.842	0.873	0.950
global	0.531	0.952	0.884	0.825	0.903	0.924
**F1-score**	**0.682**		**0.855**		**0.913**	

Supplementary Table 1: Comparative performance analysis of cell detection models

	Specific immune states										
Subtype	PD-L1+/TIL+		PD-L1+/TIL-		PD-L1-/TIL+		PD-L1-/TIL-		PD-L1+/-/TIL-B+		*P-value*
LUAD	218	0.16	186	0.14	392	0.30	124	0.09	405	0.31	1.517E-62****
LUSC	340	0.28	259	0.21	300	0.24	245	0.20	86	0.07	

	Specific immune states				
Subtype	PD-L1+/TIL+		Other		*P-value*
LUAD	224	0.10	2009	0.90	4.724E-20****
LUSC	340	0.20	1319	0.80	

	Specific immune states				
Subtype	PD-L1+/TIL-		Other		*P-value*
LUAD	191	0.09	2042	0.91	9.765E-12****
LUSC	259	0.16	1400	0.84	

	Specific immune states				
Subtype	PD-L1-/TIL+		Other		*P-value*
LUAD	392	0.18	1841	0.82	0.67
LUSC	300	0.18	1359	0.82	

	Specific immune states				
Subtype	PD-L1-/TIL-		Other		*P-value*
LUAD	124	0.06	2109	0.94	2.885E-22****
LUSC	245	0.15	1414	0.85	

	Specific immune states				
Subtype	PD-L1+/-/TIL-B+		Other		*P-value*
LUAD	719	0.32	1514	0.68	4.367E-94****
LUSC	86	0.05	1573	0.95	

Supplementary Table 2: Comparative analysis of immune cell and niche composition in LUAD and LUSC

Statistical analysis was performed using two-sided Chi-Square tests.

Abbreviations: LUAD, lung adenocarcinoma; LUSC, lung squamous cell carcinoma; PD-L1, programmed death-ligand 1; TIL, tumor-infiltrating lymphocyte; TIL-B, tumor-infiltrating B cell.

* = p < 0.05; ** = p < 0.01; *** = p < 0.001; **** = p < 0.0001.

	LUAD	LUSC
**UICC8 (state of the art)**	0.633 [0.541 - 0.723]	0.637 [0.540 - 0.729]
**Cellomics Density**	0.644 [0.546 - 0.738]	0.674 [0.565 - 0.773]
**Cellomics Niches**	0.665 [0.566 - 0.758]	0.692 [0.597 - 0.781]

Supplementary Table 3: Survival prediction results in c-scores

	1	2	3	4	5	6	7	8	9	10
Activated B cell	1.041	2.373	0	1.462	3.426	1.204	0.903	1.892	0.699	0.301
Activated cytotoxic T cell	2.34	4.25	0	3.817	4.403	2.407	2.365	4.033	1.949	1.079
Activated natural killer cell	2.161	2.711	0	2.441	2.999	1.342	1.519	2.375	2.199	1
B cell	3.344	4.587	1.079	3.632	5.496	4.129	2.665	3.943	3.418	2.766
CD8+ T cell	3.117	4.717	0	4.152	5.238	4.027	2.813	4.335	3.305	2.294
Carcinoma cell	5.91	5.923	6.246	6.096	4.77	3.734	5.295	5.559	6.187	3.4
Cytotoxic T cell	2.905	4.306	0	3.932	4.548	3.782	2.856	4.092	3.024	2.283
CD3 T-cell	3.788	5.089	0	4.823	5.293	5.2	3.382	4.521	4.129	3.748
Double positive T cell	1.462	3.557	0	2.708	4.257	2.942	0.954	3.092	0.778	0.903
Exhausted T cell	3.098	4.952	0	4.352	5.347	4.1	2.71	4.43	3.019	2.279
Exhausted regulatory T cell	1.919	3.982	0	3.252	4.341	2.719	1.398	3.418	0.778	0.954
Helper T cell	3.533	5.285	0	4.793	5.571	5.254	2.893	4.536	3.465	3.296
Immunosuppressing carcinoma cell	3.722	3.876	1.146	3.725	3.918	1.908	5.64	5.556	3.773	1.301
Immunosuppressive B cell	0.954	2.146	0	1.204	3.976	1.255	2.538	3.588	0.845	0
Immunosuppressive T cell	2.201	3.838	0	3.157	4.905	3.525	3.745	4.875	1.544	1.415
Immunosuppressive macrophage Type 1	3.82	3.838	0	2.918	4.529	2.671	3.96	5.037	2.633	3.594
Immunosuppressive macrophage Type 2	3.896	3.893	0	2.59	4.734	2.569	3.726	5.075	2.297	3.098
Immunosuppressive natural killer cell	1.146	1.531	0	0.778	2.799	0.699	3.359	3.646	0.845	0.301
Macrophage Type 1	4.944	5.033	0.602	4.246	5.097	4.357	3.716	4.866	4.33	5.014
Macrophage Type 2	4.76	4.975	0.778	4.045	5.223	4.357	3.457	4.761	4.012	4.436
Natural killer cell	3.835	3.99	0	3.523	4.354	3.039	3.083	3.819	3.71	2.841
Regulatory T cell	2.663	4.474	0	3.81	4.956	4.194	2.526	4.198	2.33	2.467

Supplementary Table 4: LUAD cell counts per niche, displayed on a logarithmic scale (base 10) across the entire cohort. Each row represents a cell phenotype type and each column represents a niche, with color intensity indicating relative abundance. The use of a logarithmic scale highlights proportional differences in cell distribution.

	1	2	3	4	5	6	7	8	9	10
Activated cytotoxic T cell	4.23	3.04	4.24	0	2.15	2.74	4.31	0.3	0	1.3
Activated natural killer cell	2.72	2.25	2.78	0	1.26	1.73	3.06	0.3	0	1.28
B cell	3.92	3.35	4.17	2.19	3.77	2.85	5.13	2.44	1.89	2.87
CD8+ T cell	4.16	3.29	4.38	0	3.54	2.87	5.11	2.68	0.78	2.29
Carcinoma cell	6.09	6.22	5.37	6.29	3.57	5.52	4.71	2.35	2.12	3.52
Cytotoxic T cell	4.06	3.31	4.13	0	3.45	2.9	4.47	2.83	0.3	2.3
CD3 T-cell	4.64	4.1	4.44	2.66	4.76	3.37	5.24	4.34	2.39	3.6
Double positive T cell	2.63	1.18	3.03	0	2.48	1.08	3.93	0	0	0.6
Exhausted T cell	4.5	3.47	4.55	0	3.84	2.93	5.23	2.15	0.48	2.25
Exhausted regulatory T cell	3.7	2.07	3.79	0	2.65	1.9	4.34	0	0	1.32
Helper T cell	4.56	3.47	4.44	0	4.92	3	5.35	3.01	1.34	3.21
Immunosuppressing carcinoma cell	4.26	4.03	5.5	2.91	1.59	5.97	3.1	0	0	1.84
Immunosuppressive B cell	1.81	1	3.67	0	0.78	3.03	3.41	0	0	0.95
Immunosuppressive T cell	3.36	2.05	4.74	0	3.23	3.95	4.5	1.62	0	1.84
Immunosuppressive macrophage Type 1	4.03	3.36	4.82	0	2.52	4.27	4.32	0.6	0.48	3.84
Immunosuppressive macrophage Type 2	4.04	3.07	4.82	0	2.46	3.94	4.53	0	2.29	3.5
Immunosuppressive natural killer cell	1.57	1.11	3.48	0	0.95	3.46	2.46	0	0	0.85
Macrophage Type 1	4.84	4.59	4.67	2.18	4.09	3.95	5.03	2.74	2.03	4.87
Macrophage Type 2	4.78	4.27	4.64	2.58	4.26	3.71	5.15	2.67	4.05	4.41
Natural killer cell	3.86	3.64	4.06	1.87	3.07	3.08	4.15	2.19	2.05	2.97
Regulatory T cell	4.03	2.7	4.38	0	4.07	2.85	4.88	2.73	0.48	2.57
Regulatory T cell	2.66	4.47	0	3.81	4.96	4.19	2.53	4.2	2.33	2.47

Supplementary Table 5: LUSC cell counts per niche, displayed on a logarithmic scale (base 10) across the entire cohort. Each row represents a cell phenotype type and each column represents a niche, with color intensity indicating relative abundance. The use of a logarithmic scale highlights proportional differences in cell distribution.
