# Supplementary material for: AI-powered spatial cell phenomics enhances risk stratification in non-small cell lung cancer
Source: Nat Commun. 2025 Nov 3;16:9701. doi: 10.1038/s41467-025-65783-z (PMC12583542; doi:10.1038/s41467-025-65783-z)
Supplement: Supplementary file 2 — Description of Additional Supplementary Information [file 41467_2025_65783_MOESM2_ESM.pdf]

Description of Additional Supplementary Files

File Name: Supplementary Data 1
Description: Clinico-pathological characteristics of the lung cohort.

File Name: Supplementary Data 2
Description: Cell phenotype definition.

File Name: Supplementary Data 3
Description: LUAD: Phenotypes per cluster, mean density_log.

File Name: Supplementary Data 4
Description: LUAD: Phenotypes per cluster, mean density.

File Name: Supplementary Data 5
Description: LUSC: Phenotypes per cluster, mean density_log.

File Name: Supplementary Data 6
Description: LUSC: Phenotypes per cluster, mean density.

File Name: Supplementary Data 7
Description: Mutational landscape of the lung cohort.
