# Supplementary material for: AI-powered spatial cell phenomics enhances risk stratification in non-small cell lung cancer
Source: Nat Commun. 2025 Nov 3;16:9701. doi: 10.1038/s41467-025-65783-z (PMC12583542; doi:10.1038/s41467-025-65783-z)
Supplement: Supplementary file 4 — Reporting Summary [file 41467_2025_65783_MOESM4_ESM.pdf]

nature portfolio

Corresponding author(s): Frederick Klauschen, Klaus-Robert Müller

Last updated by author(s): 15-10-2025

Reporting Summary

Nature Portfolio wishes to improve the reproducibility of the work that we publish. This form provides structure for consistency and transparency in reporting. For further information on Nature Portfolio policies, see our Editorial Policies and the Editorial Policy Checklist.

Statistics

For all statistical analyses, confirm that the following items are present in the figure legend, table legend, main text, or Methods section.

n/a	Confirmed	
☐	☒	The exact sample size (*n*) for each experimental group/condition, given as a discrete number and unit of measurement
☐	☒	A statement on whether measurements were taken from distinct samples or whether the same sample was measured repeatedly
☐	☒	The statistical test(s) used AND whether they are one- or two-sided<br>*Only common tests should be described solely by name; describe more complex techniques in the Methods section.*
☐	☒	A description of all covariates tested
☐	☒	A description of any assumptions or corrections, such as tests of normality and adjustment for multiple comparisons
☐	☒	A full description of the statistical parameters including central tendency (e.g. means) or other basic estimates (e.g. regression coefficient) AND variation (e.g. standard deviation) or associated estimates of uncertainty (e.g. confidence intervals)
☐	☒	For null hypothesis testing, the test statistic (e.g. *F*, *t*, *r*) with confidence intervals, effect sizes, degrees of freedom and *P* value noted<br>*Give P values as exact values whenever suitable.*
☒	☐	For Bayesian analysis, information on the choice of priors and Markov chain Monte Carlo settings
☒	☐	For hierarchical and complex designs, identification of the appropriate level for tests and full reporting of outcomes
☐	☒	Estimates of effect sizes (e.g. Cohen's *d*, Pearson's *r*), indicating how they were calculated

Our web collection on statistics for biologists contains articles on many of the points above.

Software and code

Policy information about availability of computer code

Data collection	Data collection was performed using in-house developed models for cell detection, segmentation, classification, and survival prediction. No external open-source software was used for these steps.
Data analysis	Analyses were performed in Python 3.11.9 with pandas 2.3.2, numpy 2.3.2, scipy 1.16.2, lifelines 0.30.0, scikit-learn 1.7.2, and seaborn 0.13.2. Cellular graphics were prepared using Inkscape 1.3.2. Code is available at https://github.com/gabrieldernbach/cell_niches.

For manuscripts utilizing custom algorithms or software that are central to the research but not yet described in published literature, software must be made available to editors and reviewers. We strongly encourage code deposition in a community repository (e.g. GitHub). See the Nature Portfolio guidelines for submitting code & software for further information.

Data

Policy information about availability of data

All manuscripts must include a data availability statement. This statement should provide the following information, where applicable:

- Accession codes, unique identifiers, or web links for publicly available datasets
- A description of any restrictions on data availability
- For clinical datasets or third party data, please ensure that the statement adheres to our policy

All processed cell-classification results and niche annotations have been deposited in Zenodo under accession code DOI: 10.5281/zenodo.11395885. High-resolution image tiles for each patient, including all multiplex immunofluorescence (mIF) channels and H&E (14,000 images) have been deposited in Zenodo under accession code DOI: 10.5281/zenodo.16882468. The complete raw dataset (>1 TB), including patient metadata and full image files, is available under restricted access due to

size and privacy considerations. Access can be requested from the corresponding author and will be granted within one week upon submission of a signed non-commercial use declaration and verification of an academic email address. All bona fide academic requests will be approved. No publicly available datasets were reused in this study.

Research involving human participants, their data, or biological material

Policy information about studies with human participants or human data. See also policy information about sex, gender (identity/presentation), and sexual orientation and race, ethnicity and racism.

Reporting on sex and gender	Sex and/or gender are reported as demographic variables in the reporting summary. The study collected participant sex as a biological variable
Reporting on race, ethnicity, or other socially relevant groupings	Race and ethnicity data were not collected in this study, as these variables are not routinely recorded in European Union clinical practice due to data protection regulations and ethical considerations
Population characteristics	The study population comprised all patients with histologically confirmed lung cancer who had undergone surgical resection and survived at least 15 years post-operatively. Eligible participants were identified from the institutional cancer registry and included patients who underwent resection
Recruitment	All lung cancer patients of the 15 years in registered in the institutions.
Ethics oversight	The study was performed according to the ethical principles for medical research of the Declaration of Helsinki and was approved by the Ethics Committee of the Charité University Medical Department in Berlin (EA4/082/22). All patients provided written informed consent for the scientific use of their archived tissue and associated clinical data. The study was retrospective and involved no additional procedures or interventions; therefore, no participant compensation was provided. Mutation status, recurrence information and smoking status were incomplete at the time of writing.

Note that full information on the approval of the study protocol must also be provided in the manuscript.

Field-specific reporting

Please select the one below that is the best fit for your research. If you are not sure, read the appropriate sections before making your selection.

[x] Life sciences [ ] Behavioural & social sciences [ ] Ecological, evolutionary & environmental sciences

For a reference copy of the document with all sections, see nature.com/documents/nr-reporting-summary-flat.pdf

Life sciences study design

All studies must disclose on these points even when the disclosure is negative.

Sample size

The initial cohort comprised 1,168 patients. Sample size determination for each experiment/figure and rationale are described in detail in the Methods section, in Figure 1A, and Supplementary Figure 11. For survival analysis, patients were excluded if complete tumor removal could not be confirmed (R status = 1), if observed or censored survival was less than three months (to avoid bias from perioperative mortality), if adjuvant therapy introduced potential distributional shifts, or if they presented with late-stage disease (UICC8 stage 4), as the main analysis focused on early-stage patients. Patients who underwent adjuvant therapy or had late-stage disease were analyzed separately. In addition, propensity score matching was implemented in LUAD to balance patient characteristics between cohorts, increasing comparability and reliability of results. Although no formal statistical sample size calculation was performed, the cohort size of 1,168 patients from two independent cancer centers provides sufficient statistical power for robust subgroup analyses and validation. The sample size is large compared to previous studies and representative of real-world clinical practice.
Detailed demographic information (number of patients, sex, mean and median age) is provided below for each experiment/ figure:

Figures 2A, 2E, 3L, 3N, 5A: 2,233 tumor samples from 663 patients (303 female, 360 male; mean age = 64.7 years; median age = 64.7 years).
Figures 2B, 2G, 4L, 4N, 5B: 1,657 tumor samples from 462 patients (116 female, 346 male; mean age = 67.8 years; median age = 68.7 years).
Figures 6A-C: 109 patients (45 female, 64 male; mean age = 64.7 years; median age = 65.4 years).
Figure 6D: RS1 n = 61 patients (29 female, 32 male; mean age = 64.3 years; median age = 64.0 years); RS2+3 n = 163 patients (64 female, 99 male; mean age = 65.8 years; median age = 66.1 years); UICC I n = 148 patients (66 female, 82 male; mean age = 65.2 years; median age = 64.8 years).
Figures 7A-C: n = 109 patients (24 female, 85 male; mean age = 69.9 years; median age = 70.4 years).
Figure 7D: RS1 n = 66 patients (20 female, 46 male; mean age = 69.0 years; median age = 69.9 years); RS2+3 n = 209 patients (54 female, 155 male; mean age = 69.3 years; median age = 70.3 years); UICC I n = 163 patients (49 female, 114 male; mean age = 69.3 years; median age = 70.2 years).
Figure 8D: n = 10 patients (6 female, 4 male; mean age = 68.1 years; median age = 68.0 years).
Figure 8H: n = 10 patients (3 female, 7 male; mean age = 69.8 years; median age = 74.0 years).
Supplementary Figures 6A: n = 769 patients (301 female, 468 male; mean age = 65.7 years; median age = 66.3 years).
Supplementary Figures 7A: 3,807 tumor samples from 1125 patients (419 female, 706 male; mean age = 66.0 years; median age = 66.3 years).
Supplementary Figures 12A+B: n = 109 patients (45 female, 64 male; mean age = 64.7 years; median age = 65.4 years).
Supplementary Figures 13A+B: n = 109 patients (24 female, 85 male; mean age = 69.9 years; median age = 70.4 years).
Supplementary Figure 14A: n = 72 patients (39 female, 33 male; mean age = 62.5 years; median age = 61.6 years).
Supplementary Figure 14B: n = 18 patients (6 female, 12 male; mean age = 68.0 years; median age = 68.8 years).

Supplementary Figure 15A: n = 164 patients (75 female, 89 male; mean age = 64.4 years; median age = 64.1 years).
Supplementary Figure 15B: n = 98 patients (18 female, 80 male; mean age = 64.1 years; median age = 63.6 years).
Supplementary Figure 16B: n = 147 patients (63 female, 84 male; mean age = 65.4 years; median age = 65.9 years).
Supplementary Figure 16C: n = 178 patients (42 female, 136 male; mean age = 69.6 years; median age = 71.0 years).
Supplementary Figure 17A+B: n = 224 patients (93 female, 131 male; mean age = 65.4 years; median age = 65.7 years).
Supplementary Figure 17C: n = 194 patients (85 female, 109 male; mean age = 65.1 years; median age = 65.4 years).
Supplementary Figure 17D: n = 131 patients (58 female, 73 male; mean age = 65.8 years; median age = 65.9 years).
Supplementary Figure 17E: n = 211 patients (88 female, 123 male; mean age = 65.7 years; median age = 65.7 years).
Supplementary Figure 17F: n = 180 patients (70 female, 110 male; mean age = 65.4 years; median age = 65.8 years).
Supplementary Figure 17G: n = 216 patients (90 female, 126 male; mean age = 65.5 years; median age = 65.7 years).
Supplementary Figures 17H+I: n = 275 patients (74 female, 201 male; mean age = 69.2 years; median age = 70.3 years).
Supplementary Figure 17J: n = 234 patients (61 female, 173 male; mean age = 69.3 years; median age = 70.5 years).
Supplementary Figure 17K: n = 156 patients (46 female, 110 male; mean age = 68.6 years; median age = 69.9 years).
Supplementary Figure 17L: n = 256 patients (67 female, 189 male; mean age = 69.3 years; median age = 70.3 years).
Supplementary Figure 17M: n = 270 patients (71 female, 199 male; mean age = 69.2 years; median age = 70.3 years).
Supplementary Figures 18C-F: 2,189 tumor samples from 663 patients (303 female, 360 male; mean age = 64.7 years; median age = 64.7 years).
Supplementary Figures 18G+H: n = 108 patients (44 female, 64 male; mean age = 64.7 years; median age = 65.4 years).
Supplementary Figures 19C-F: 1,618 tumor samples from 462 patients (116 female, 346 male; mean age = 67.8 years; median age = 68.7 years).
Supplementary Figures 19G+H: 109 patients (24 female, 85 male; mean age = 69.9 years; median age = 70.4 years).
Supplementary Figure 20A+C (LUAD part)+D+F: 2,233 tumor samples from 663 patients (303 female, 360 male; mean age = 64.7 years; median age = 64.7 years).
Supplementary Figure 20B+C (LUSC part)+E+G: 1,657 tumor samples from 462 patients (116 female, 346 male; mean age = 67.8 years; median age = 68.7 years).
Supplementary Figures 23A+B: n = 10 patients (6 female, 4 male; mean age = 68.1 years; median age = 68.0 years).
Supplementary Figures 23C+D: n = 10 patients (3 female, 7 male; mean age = 69.8 years; median age = 74.0 years).
All numbers are also reported in the Methods and in the Figure legends for full transparency.

Data exclusions

Approximately 10% of tissue spots were excluded due to tissue damage and tears during staining; spots with less than 0.7 square millimeters of aligned tissue or more than 3 square millimeters were rejected; 22 patients with mixed subtype (ASC) were also excluded from analysis. In the subgroup analyses, patients were further excluded if complete tumor removal could not be confirmed (R status = 1) and if observed or censored survival was less than three months. A detailed overview of all exclusion criteria is provided in Supplementary Figure 11.

Replication

The model was trained on the Berlin cohort and independently validated on the Cologne cohort , demonstrating successful replication with improved survival prediction beyond UICC8 staging in both LUAD and LUSC subtypes.

Randomization

This was a retrospective observational study using existing patient tissue samples and clinical data, so randomization was not applicable to the study design.

Blinding

Given the retrospective nature and use of automated AI analysis on existing tissue samples, traditional blinding was not relevant to the study design.

Reporting for specific materials, systems and methods

We require information from authors about some types of materials, experimental systems and methods used in many studies. Here, indicate whether each material, system or method listed is relevant to your study. If you are not sure if a list item applies to your research, read the appropriate section before selecting a response.

Materials & experimental systems

n/a	Involved in the study	
☐	☒	Antibodies
☒	☐	Eukaryotic cell lines
☒	☐	Palaeontology and archaeology
☒	☐	Animals and other organisms
☐	☒	Clinical data
☒	☐	Dual use research of concern
☒	☐	Plants

Methods

n/a	Involved in the study	
☒	☐	ChIP-seq
☒	☐	Flow cytometry
☒	☐	MRI-based neuroimaging

Antibodies

Antibodies used

Anti-human CD3 (BioCare ACI 3170 A), CD4 (Abcam ab238798), CD8 (BioCare ACI3160CF), CD20 (Thermo/eBio 14-0202-82), CD56 (Thermo/eBio 701379), CD68 (BioCare CM033CF), CD163 (Abcam ab213612), FoxP3 (Thermo/eBio 14-4777-82), PanCK (Bethyl/Fortis PGS170523), PD-1 (Abcam ab251613), PD-L1 (Abcam ab226766), and Granzyme B (Abcam ab214443).
All antibodies were provided in pre-optimized Ultivue kits and were diluted 1:100 in antibody diluent according to the manufacturer's

	standard protocol. The staining was performed by Ultivue as a standardized service. Antibody targets, suppliers, catalog numbers, and dilutions are listed in the Methods section of the manuscript. Antibody dilutions and staining protocols are proprietary to Ultivue and not disclosed, as the assay is delivered as a service product. Further details can be obtained from Ultivue (https://ultivue.com).
Validation	Validation of all primary antibodies was performed by Ultivue as part of their standardized multiplex immunofluorescence service. Antibodies were validated on tonsil and healthy lung tissue as a positive and negative control respectively. Additional details can be obtained from Ultivue (https://ultivue.com).<br>Further PD-L1 validation was performed by comparing multiplex immunofluorescence results with orthogonal immunohistochemistry scoring by expert pathologists; lymphocyte counts were validated by comparing mIF-derived CD3/CD20-positive cells with H&E-based quantification using a foundation model.

Clinical data

Policy information about clinical studies

All manuscripts should comply with the ICMJE guidelines for publication of clinical research and a completed CONSORT checklist must be included with all submissions.

Clinical trial registration	Not applicable as this was a retrospective observational study using existing patient tissue samples and clinical data from 2006-2019
Study protocol	The study protocol involved tissue microarray construction, 12-plex immunofluorescence staining, H&E staining, image registration, AI-based analysis pipeline development, and survival modeling on retrospectively collected samples
Data collection	Data was collected from 1,168 NSCLC patients treated at University Hospital Cologne and Charité Berlin between 2006-2019; 4 tissue cores per patient were assembled into tissue microarrays and subjected to multiplex immunofluorescence imaging.
Outcomes	Primary outcome was overall survival; secondary outcomes included risk stratification improvement beyond UICC8 staging and identification of spatial cell niches associated with survival, measured using concordance index (c-index) and Kaplan-Meier survival analysis.

Plants

Seed stocks	Not applicable - this is a human clinical study, not a plant study.
Novel plant genotypes	Not applicable - this is a human clinical study, not a plant study
Authentication	Not applicable - this is a human clinical study, not a plant study
